# Structural and functional insight into the *Mycobacterium tuberculosis* protein PrpR reveals a novel type of transcription factor

**DOI:** 10.1093/nar/gkz724

**Published:** 2019-08-26

**Authors:** Su Tang, Nathan D Hicks, Yu-Shan Cheng, Andres Silva, Sarah M Fortune, James C Sacchettini

**Affiliations:** 1 Department of Biochemistry and Biophysics, Texas A&M University, College Station, TX 77840, USA; 2 Department of Immunology and Infectious Diseases, Harvard T.H. Chan School of Public Health, Boston, MA 02115, USA; 3 Department of Chemistry, Texas A&M University, College Station, TX 77840, USA; 4 Ragon Institute of MGH, MIT, and Harvard, Cambridge, MA 02139, USA

## Abstract

The pathogenicity of *Mycobacterium tuberculosis* depends upon its ability to catabolize host cholesterol. Upregulation of the methylcitrate cycle (MCC) is required to assimilate and detoxify propionyl-CoA, a cholesterol degradation product. The transcription of key genes *prpC* and *prpD* in MCC is activated by MtPrpR, a member of a family of prokaryotic transcription factors whose structures and modes of action have not been clearly defined. We show that MtPrpR has a novel overall structure and directly binds to CoA or short-chain acyl-CoA derivatives to form a homotetramer that covers the binding cavity and locks CoA tightly inside the protein. The regulation of this process involves a [4Fe4S] cluster located close to the CoA-binding cavity on a neighboring chain. Mutations in the [4Fe4S] cluster binding residues rendered MtPrpR incapable of regulating MCC gene transcription. The structure of MtPrpR without the [4Fe4S] cluster-binding region shows a conformational change that prohibits CoA binding. The stability of this cluster means it is unlikely a redox sensor but may function by sensing ambient iron levels. These results provide mechanistic insights into this family of critical transcription factors who share similar structures and regulate gene transcription using a combination of acyl-CoAs and [4Fe4S] cluster.

## INTRODUCTION

*Mycobacterium tuberculosis* (*Mtb*) caused an estimate of 1.6 million deaths in 2017 alone, and has developed resistance to many commonly used antibiotics (1). Part of *Mtb*’s effectiveness as a pathogen is that it can use fatty acids and cholesterol as primary nutrient sources during infection (2–4). The degradation of both odd-chain fatty acids and cholesterol produces propionyl coenzyme A (propionyl-CoA) (5–9) which must be further metabolized as accumulation of propionyl-CoA leads to the toxicity to the bacilli (10,11).

One of the major pathways to assimilate propionyl-CoA is the methylcitrate cycle (MCC) (Supplementary Figure S1). The key enzymes of the MCC are typically clustered in the propionate metabolic operon (*prp* operon), which includes methylcitrate synthase (MCS, also named PrpC), methylcitrate dehydratase (MCD, also named PrpD) and methylisocitrate lyase (MCL, also named PrpB) (10,12). The *prp* operon of *Mtb* only contains two genes *prpC* (*rv1131*) and *prpD* (*rv1130*). The *Mtb* genome does not encode a functionally unique MCL, but instead employs isocitrate lyase 1 (Icl1), an enzyme in the anaplerotic glyoxylate shunt (11–13), to accomplish the MCL function. Certain strains of *Mtb*, including the clinical strain CDC1551, also contain a second copy of Icl (Icl2), which has minimal MCL activity (12).

**Figure 1.**
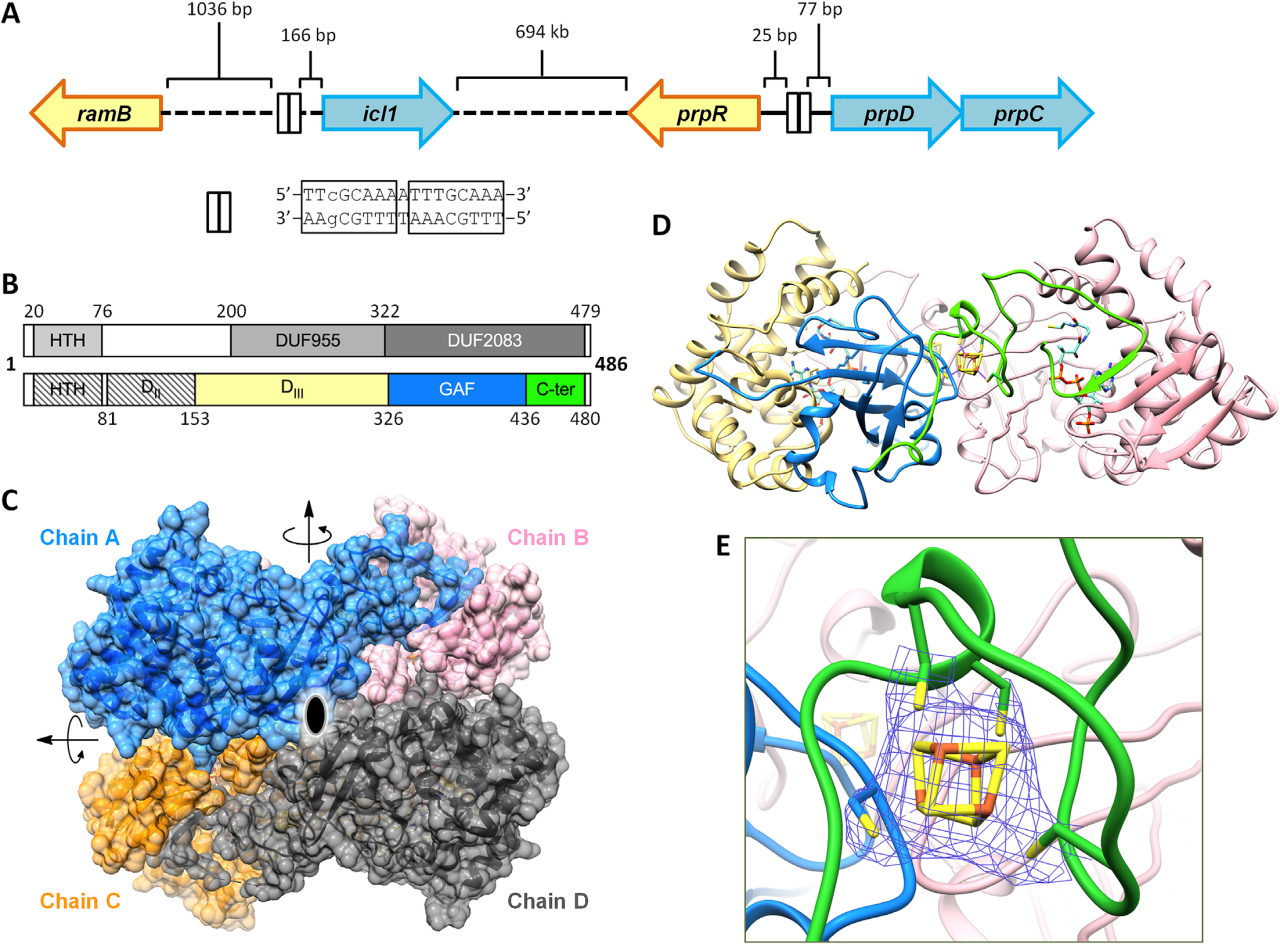
Genome organization of *ramB/icl1* and *prp* regulons and structure of MtPrpR. (**A**) Genome organization of *ramB-icl1* and *prpR-prpDC* regulons. Dashed lines indicate the presence of genes in between. Distances of the intergenic regions are indicated. The tandem repeats are represented as boxes. (**B**) Domain organization of MtPrpR: gray-scaled boxes and the numbers above indicate the predicted domain borders; color-filled boxes and the numbers below indicate the domains observed in the structure. The D_III_ domain is colored in light yellow; the GAF-like domain is colored in blue; the C-terminal region is colored in green. The hatched boxes indicate the domains that were either truncated (HTH) from or not visible (D_II_) in the protein construct. (**C**) Crystal structure of the MtPrpR_81–486_ tetramer. The four chains are shown in different colors. Arrows and the central ellipse indicate the 2-fold rotational relationships between each pair of polypeptide chains. (**D**) The top half of the MtPrpR tetramer. Chain A is colored by domain organization in the same way as in B; Chain B is colored in pink. (**E**) Close up of the [4Fe4S] cluster-binding site. The 2mFo-DFc electron density of the cluster is contoured at 1 σ.

In *Mtb*, the transcription of the *prp* operon is stringently controlled by a gene-specific transcription factor, PrpR (propionate regulator, Rv1129c, MtPrpR) (8,14,15). The *prpR* gene is adjacent to the *prp* operon, but it runs in the opposite direction (Figure 1A). MtPrpR has been shown to activate transcription of both itself and the *prp* operon in response to propionate or cholesterol (8,15). Deleting *prpR* from the *Mtb* genome renders the bacterium incapable of activating the *prp* operon in response to propionate or cholesterol *in vitro* and eliminates its ability to grow on media containing these molecules as sole carbon sources (8,15). However, *prpR* deficient mutants are prevalent in isoniazid- and other drug-resistant *Mtb* clinical isolates (16,17), possibly because *prpR* mutations cause a defect in the MCC and slow down bacterial metabolism during infection, leading to drug tolerance (17).

There is little structural information available for any homolog of MtPrpR, which makes it difficult to identify a common regulatory mechanism for this family of transcription factors. For example, we still do not understand how MtPrpR senses signaling molecules, beyond previous studies suggesting that 2-methylcitrate, an intermediate of MCC, was a PrpR coactivator in Corynebacterium glutamicum (18). It has also been suggested that cyclic AMP (cAMP) is indirectly involved in MCC modulation (19,20). Conserved domain analysis (21) was only able to annotate the N-terminus of MtPrpR as the helix-turn-helix DNA binding domain (Figure 1B). The rest of the protein was previously annotated as two domains of unknown function, DUF955 and DUF2083 (14). DUF955 was recently updated to be an IrrE N-terminal-like domain, which was reported as a Zn-dependent metaloprotease (22,23).

The *Mtb* genome also encodes an MtPrpR paralog, MtRamB (regulator of acetate metabolism B, Rv0465c), which shares 54% protein sequence identity with MtPrpR and is located upstream of *icl1* (Figure 1A). Unlike MtPrpR, which is largely accepted as a transcriptional activator (8,14,15), MtRamB is more likely to be a transcriptional repressor in the presence of fermentable carbon sources such as dextrose (14,24).

A 17 bp DNA sequence between *prpR* and *prpD* has been proposed as the primary recognition sequence of MtPrpR (15) (Figure 1A). It consists of a perfect (TTTGCAAA) and an imperfect (TTTGCgAA) palindrome separated by one base pair. The exact 17 bp DNA sequence also exists upstream of the *icl1* gene, but is not found elsewhere in the *Mtb* genome. A DNA fragment containing TTTGC(A/G)AA was also mapped to MtRamB binding sites (24–26). The similarity of the recognition sequences suggests the possibility of crosstalk between MtPrpR and MtRamB during transcriptional regulation. To unravel the potential structure-function relationship of MtPrpR and its homologs, we solved the crystal structures of different forms of MtPrpR. We show that MtPrpR is a tetrameric transcription factor. The C-terminal region of the protein binds to an iron-sulfur cluster, which enables the protein to bind Coenzyme A and acetyl-CoA molecules tightly as part of the regulatory mechanism. The structure of MtPrpR represents the first structure of this family of transcription factors that control several resolved and unresolved bacterial carbon metabolic pathways.

## MATERIALS AND METHODS

### Bacterial strains, media and growth conditions

*Mtb* strains were maintained in complete 7H9 media (Middlebrook 7H9 salts supplemented with 0.2% glycerol, 0.05% Tween-80 and 10% Middlebrook OADC). The *prpR* deletion mutant of *Mtb* H37Rv (H37RvΔ*prpR*) was obtained from the Sassetti lab as described previously (8). *PrpR* deletion mutants carrying a rescuing wild-type or mutant allele were then constructed as described previously (17). Briefly, a kanamycin resistant plasmid carrying the *prpR* wild-type or mutant gene and 95 bp of the sequence upstream of the proposed translational start site was integrated at the L5 phage integration site of the *prpR* deletion strain.

The knockdown of *ramB* was performed using the CRISPR interference (CRISPRi) system optimized for *Mtb* (27) targeting the sequence GCGGGCAGGTGCCGCCGCTGGA within the *ramB* ORF. The anhydrous tetracycline (ATc) inducible *ramB* knockdown vector was transformed into both wild-type H37Rv and the H37RvΔ*prpR* mutant strains.

### Protein sequence alignment and analysis

MtPrpR and a group of representative homologous protein sequences were aligned in Clustal Omega (28). The output alignment file was further analyzed and illustrated using ESPpript 3.0 (29). The proteins with names are those whose functions are either previously reported or recognizable based on the genome context. The proteins with question marks are those with unknown functions and adjacent to uncharacterized genes or operons. Protein domain annotation was performed using InterPro (21). Protein secondary structure was predicted by PSIPRED (30).

### Cloning, protein expression and purification

The *Mtb* gene *rv1129c* (*prpR*) encoding the full-length PrpR protein (MtPrpR_M1) was cloned as previously described (15). Briefly, the gene was amplified from the *Mtb* H37Rv genome using PrpR_M1_Fw and PrpR_Rv primers. (All primers were synthesized by Integrated DNA Technologies, and are listed in Supplementary Table S1.) The polymerase chain reaction (PCR) product was treated with the BamHI and XhoI restriction enzymes (New England Biolabs) and cloned into a pET28a vector (Novagen).

A reannotation of the *Mtb prpR* ORF (31) suggested that the ninth amino acid valine might be the translational start site. A *PrpR*_V9 construct removing the first eight amino acids from the N-terminus was subcloned into a pET28a vector between the BamHI and XhoI sites using PrpR_V9_Fw and PrpR_Rv primers. Both MtPrpR_M1 and MtPrpR_V9 were expressed in *Escherichia coli* BL21(DE3). The cells were grown in LB media supplemented with 50 μg ml^−1^ kanamycin to an OD_600_ of 0.8, induced with 0.08 mM Isopropyl β-D-1-thiogalactopyranoside (IPTG) and shaken at 18°C for another 12–16 h before being pelleted. The cells were resuspended in lysis buffer (100 mM Na_2_HPO_4_/NaH_2_PO_4_ pH 7.5, 200 mM NaCl, 30 mM imidazole), homogenized using a Microfluidizer M-100P (Microfluidics). Cell debris was clarified and the supernatant was applied to Ni-NTA resin (Qiagen). The resin was washed with 20 column volumes of wash buffer (100 mM Na_2_HPO_4_/NaH_2_PO_4_ pH 7.5, 200 mM NaCl, 60 mM imidazole). An isocratic elution was performed using elution buffer (100 mM Na_2_HPO_4_/NaH_2_PO_4_ pH 7.5, 200 mM NaCl, 300 mM imidazole).

A *prpR*_81–486_ construct that truncated the N-terminal helix-turn-helix domain was subcloned into a ligation-independent cloning (LIC) vector pMCSG7 (32) with primers PrpR_D81_Fw and PrpR_V486_Rv. This construct contains a His_6_-tag and a TEV cleavage site to the N-terminus of the target protein. The plasmid was transformed into *E. coli* BL21(DE3) competent cells. The cells were grown at 37°C in LB medium supplemented with 100 μg ml^−1^ carbenicillin to an OD_600_ of 0.8. Protein expression was induced by 0.3 mM IPTG. Cells were grown at 18°C for another 12–16 h before being pelleted. Protein purification was analogous to that of the full-length proteins except that 20 mM Tris–HCl pH7.8 was used instead of 100 mM Na_2_HPO_4_/NaH_2_PO_4_ pH 7.5 in all the buffers. The salt and imidazole concentrations remained unchanged. The purified MtPrpR_81–486_ was about 95% pure according to sodium dodecyl sulphate-polyacrylamide gelelectrophoresis (SDS-PAGE) analysis.

The protein was diluted in IEX buffer A (20 mM Tris–HCl pH 7.8) until the NaCl concentration was below 100 mM and was loaded to an anion exchange HiTrap Q FF column (GE Healthcare). The column was washed for 10 column volumes by mixing 80% IEX buffer A with 20% IEX buffer B (20 mM Tris–HCl pH 7.8, 1.0 M NaCl). An isocratic elution was performed with 30% IEX buffer B. N-terminal His_6_-tagged protein was concentrated immediately to ∼30 mg ml^−1^ and applied onto a HiLoad 26/600 Superdex-200 column (GE Healthcare) equilibrated with size exclusion chromatography (SEC) buffer (20 mM Tris–HCl pH 7.8, 100 mM NaCl, 3 mM dithiothreitol (DTT)). Peak fractions were pooled and concentrated to 24 mg ml^−1^ for crystallization trials.

MtPrpR_81–486_ point mutations were introduced using the primers listed in Supplementary Table S1 and the QuikChange site-directed mutagenesis kit (Agilenet). The F155H variant was expressed and purified following the same procedure for the wild-type protein.

Selenomethionine (Se-Met) incorporation was adapted from previous literature (33). Specifically, Se-Met-MtPrpR_81–486_ was expressed by growing the bacteria in M9 minimal salt media supplemented with 0.4% glucose, 2 mM MgSO_4_, 0.1 mM CaCl_2_ and 100 μg ml^−1^ carbenicillin. Cells were grown to an OD_600_ of 0.6 and chilled on ice. A total of 100 mg l^−1^ each of L-lysine, L-threonine, L-phenylalanine, 50 mg l^−1^ each of L-leucine, L-isoleucine, L-valine and 60 mg l^−1^ L-selenomethionine, were added before induction along with 100 mg l^−1^ ferrous ammonium sulfate. Cells were shaken at 18°C for 15 min, and protein expression was induced with 0.3 mM IPTG for 12–16 h. The selenomethionine-derived protein purification procedure was identical to that of the native protein and its behavior was also similar to that of the native protein.

Transition metal ion evaluations were performed by growing the *E. coli* cells in M9 minimal salt media supplemented with 0.4% glucose, 2 mM MgSO_4_, 0.1 mM CaCl_2_ and 100 μg ml^−1^ carbenicillin to an OD_600_ of 0.8. Each flask was supplied with 25 μM one of the chloride salts of Fe^3+^, Co^2+^, Ni^2+^, Cu^2+^ and Zn^2+^ 15 min before induction. Protein expression was induced with 0.3 mM IPTG for 16 h at 18°C. The total protein expression in the whole cell lysate and the soluble protein in the supernatant were analyzed using SDS-PAGE.

*PrpR*_155–440_ was subcloned into a pMCSG7 vector using primers PrpR_F155_Fw and PrpR_E440_Rv. Procedures for protein expression and affinity purification were similar to those of MtPrpR_81–486_ except that all purification buffers contain 500 mM NaCl. The protein was dialyzed overnight against 20 mM Tris–HCl pH7.8, 500 mM NaCl while the His_6_-tag was cleaved with 0.02 mg ml^−1^ TEV protease. The TEV protease was expressed and purified from plasmid pMHTDelta238 (DNASU Plasmid Repository) as described (34). The protein was reapplied to the Ni-NTA resin to remove the His_6_-tag and the residual impurities. The protein was concentrated to about 10 mg ml^−1^ and injected to a HiLoad 26/600 Superdex-75 column (GE Healthcare) pre-equilibrated with 20 mM Tris–HCl pH 7.8, and 400 mM NaCl. The peak fractions were pooled and concentrated to ∼10 mg ml^−1^.

Analytical SEC for each protein construct was performed using the Superdex 200 10/300 GL (GE Healthcare). SEC buffer for each protein construct was identical to their respective preparative SEC buffer.

### Crystallization

Freshly purified N-terminal His-tagged MtPrpR_81–486_ or MtPrpR_81–486__F155H variant proteins at 24 mg ml^−1^ was screened against over 900 crystallization conditions using sitting-drop vapor diffusion set by a Mosquito Crystal liquid handler (TTP Labtech Inc). Protein was equilibrated with the crystallization conditions in 17°C. Brown color crystals appeared within 2 days. The best growth condition contained either 100 mM HEPES pH 7.5 or 100 mM Bicine pH 9.5, 1.0 M sodium acetate, 0.08 mM Zwittergent 3–14.

MtPrpR_155–440_ protein with the His-tag cleaved was screened against over 900 conditions at 10 mg ml^−1^ in the presence and absence of 1 mM CoA. Crystals of identical morphology for both formulations appeared in about one week in 0.2 M CaCl_2_•2H_2_O, 0.1 M HEPES pH 7.5, 28% v/v PEG 400.

### Data collection and structure determination

Crystal X-ray diffraction data were collected at Beamline 19-ID, 19-BM, or 23-ID at the Advance Photon Source, Argonne National Laboratory in Chicago. Anomalous scattering for selenomethionine derived MtPrpR_81–486_ (PDB code: 6CZ6) was collected around the peak position at 0.97949 Å. (We planned for Fe phasing, but the beam at low energy was not stable and we succeeded with Se phasing, thus, we did not perform Fe phasing.) The crystal diffracted to ∼2.7 Å resolution. Crystal symmetry and diffraction intensity were analyzed with Denzo in HKL2000, scaled and reduced with Scalepack in HKL2000 (35). The selenomethionine derived crystal belonged to the P2_1_2_1_2_1_ space group with a = 96.19 Å, b = 142.37 Å, c = 145.97 Å, α = β = γ = 90° and contained one biological tetramer per asymmetric unit (ASU) in D2 symmetry. Experimental phasing was achieved using single-wavelength anomalous diffraction (SAD). Heavy atom sites were searched in SHELXD (36) (SHELXC/D/E pipeline in CCP4 (37)). Seventeen out of 28 possible heavy atom sites were found, all of which were correct when compared to the final structure. The electron density map was calculated with SHELXE (38), followed by density modification with Parrot (39) in CCP4 by applying non-crystallographic symmetry (NCS) calculated from the heavy atom substructure. Automated initial model building plus refinement was conducted using Buccaneer (40) in CCP4. Manual model building was performed in Coot (41). The [4Fe4S] cluster and Coenzyme A were built into the electron density in all four chains in the ASU.

The native PrpR_81–486_ crystal (PDB code: 6CYY) was collected to 2.5 Å and had slightly different unit cell dimensions. The crystal was indexed to a higher symmetry of the P4_1_2_1_2 space group with a = b = 144.88 Å, c = 97.77 Å. In this case, a biological tetramer was shared by two ASUs, each containing two polypeptide chains. Since the native crystal was non-isomorphous to the heavy atom derived one, a molecular replacement search was performed in Phaser-MR (42) in PHENIX (43) using one polypeptide chain from the Se-PrpR_81–486_ tetramer. Refinement was carried out using phenix.refine (44), by randomizing the B-factors of the MR solution and performing simulated annealing to remove as much as possible of the R-free bias introduced by previous refinement. Real space refinement was performed in phenix.refine and Coot. Fifteen Translation/Libration/Screw (TLS) groups were defined by phenix.refine and applied in the refinement. X-ray/stereochemistry and X-ray/ADP weights were optimized in the final cycles of refinement to improve the refinement statistics.

The MtPrpR_81–486__F155H variant crystal (PDB code: 6CYJ) diffracted to 2.7 Å, and the unit cell was isomorphous to the native MtPrpR_81–486_. The structure of the wild-type protein was directly refined against the mutant data by transferring the test set from the wild-type data. The process of refinement was analogous to that of the wild-type protein.

The MtPrpR_155–440_ crystal (PDB code: 6D2S) diffracted to 1.8 Å and belonged to the I222 space group with a = 75.03, b = 81.71, c = 95.37, α = β = γ = 90°. A molecular replacement search was performed in Phaser-MR using one molecule of MtPrpR_81–486_ trimmed to appropriate positions as the search model. The manual model building and refinement were performed in Coot and phenix.refine analogous to that of the native MtPrpR_81–486_.

The interface analysis was performed using PISA (45). The 3D structure similarity search was performed in Dali (46). The structure figures were generated using UCSF Chimera (47).

### [4Fe4S] cluster identification and characterization

The [4Fe4S] cluster was initially identified with the UV-visible spectroscopy. MtPrpR_81–486_ was diluted to 30 μM and transferred to a quartz cuvette of one cm path length. The UV-visible spectra were recorded from 200 to 800 nm using a Cary 50 UV-Vis spectrophotometer (Varian/Agilent Technologies, Santa Clara, CA, USA).

Laser Ablation Inductively Coupled Plasma Mass Spectrometry (LA-ICP-MS) was conducted using an ELAN DRC II ICP Mass Spectrometer (Perkin Elmer) to further confirm the identity and the stoichiometry of the metal ion. The iron standard was diluted in 1% HNO_3_ to 25, 50, 100, 150, 200 ppb, and plotted as a standard curve. The protein sample (10 mg ml^−1^) was denatured, diluted 200- and 400-fold in 1% HNO_3_ to 1.04 and 0.52 μM, respectively, and centrifuged to remove the protein. The supernatant was then subjected to the ICP-MS system. The iron concentration was calculated based on the standard curve.

X-band continuous wave EPR spectroscopy was performed using a Bruker EleXsys E500 EPR spectrometer. The EPR signals were recorded at 10 K. The microwave frequency was 9.38 GHz. The field modulation was 5 G at 100 KHz, and the microwave power was 0.2 mW. The MtPrpR_81–486_ sample aliquots at 200–250 μM (10–12 mg ml^−1^) were treated aerobically with 1 mM ferricyanide ion, 2 mM dithionite or 5 mM ethylenediaminetetraacetic acid (EDTA) for >3 h on ice before EPR spectra measurements.

The H_2_O_2_ oxidation test was performed by mixing 20 μM of purified MtPrpR_81–486_ with different concentrations of H_2_O_2_ (Sigma-Aldrich) in the SEC buffer. The property of [4Fe4S] cluster was monitored optically using the UV-visible spectrometry at indicated time points.

The NO response was performed as described previously (48). Briefly, PROLI NONOate (Cayman Chemical), the NO donor compound, was dissolved in 25 mM NaOH and supplemented at indicated concentrations into 50 μM MtPrpR_81–486_ in the SEC buffer. Each molecule of PROLI NONOate can release two molecules of NO (t_1/2_ = 13 s at 25°C) (49). The UV-visible spectrum was measured after a 10 min incubation.

The dithionite ion response was performed by incubating 30 μM purified MtPrpR_81–486_ with different concentrations of freshly made sodium dithionite (Sigma-Aldrich). The UV-visible spectra were monitored at indicated time points.

The impact of a chelator on the cluster and the oligomeric state of the protein was assessed by incubating 150 μM MtPrpR_81–486_ with 20 mM Tris–HCl pH 7.8, 100 mM NaCl, 3 mM DTT, 10 mM EDTA for 3 h or 36 h. The protein was then injected to a Superdex 200 10/300 analytical column equilibrated with the same buffer.

### Coenzyme A derivative isolation and identification

The CoA derivative isolation procedure was performed at 4°C to maintain the integrity of the molecules. A total of 100 mg of 25 mg ml^−1^ of the wild-type MtPrpR_81–486_ or the F155H variant was denatured in 10 volumes of 8 M urea acidified with 0.1 M formic acid to stabilize potential thioester bonds. Urea was freshly made and purified with mixed bed resin (Sigma-Aldrich) to minimize the content of isocyanate and other ions. The denatured protein solution was passed through a 30 kDa cut-off concentrator to remove the protein, and the flow-through was collected and loaded onto Q fast flow sepharose anion exchange resin (GE Healthcare Life Sciences). The resin was washed with five column volumes of 0.1 M formic acid and eluted with two column volumes of 2.0 M ammonium formate in 0.1 M formic acid. The eluate was lyophilized and dissolved in 0.2 ml pure water for identification. The LC-MS analysis was adapted from previous research on CoA derivatives. Specifically, a 10 μl sample solution was injected into a Kinetex 2.6 μm EVO C18 100A LC column 100 × 4.6 mm and separated by a mobile phase gradient elution of 50 mM ammonium acetate (A) and acetonitrile + 0.1% formic acid (B) at a flow rate of 0.5 ml min^−1^. An Agilent 1200 Series LC system was used to start (B) at 10% and increase linearly to 100% from 0 to 8 min, hold (B) at 100% from 8–12 min, decrease linearly from 100 to 10% from 12 to 14 min and held at 10% for an additional 3 min (total run time 17 min). A Bruker Daltonics micrOTOF-Q II LC-MS with an electrospray ionization system was operated in positive mode at a source temperature of 220°C, dry nitrogen gas flow of 11 l min^−1^ and under 3.5 bars of pressure. A 0.4 mg ml^−1^ sodium formate external calibration solution was directly injected at 13 min for post data analysis and calibration. Quantification was based on integrating peak area corresponding to the elution of the target compound in the extracted product ion chromatograms. Data was processed using Bruker Hystar Software 4.1 and extracted ion chromatograms were created for each target compound.

### Transcription level quantification

Quantification of gene expression was performed using reverse transcription, quantitative PCR (RT-qPCR) as described previously (17). *PrpR* variant strains were grown to log phage in 7H9 complete media, spun down and resuspended to an OD_600_ of 0.1 in 10 ml of 7H12 media (7H9 salts, 0.2% glycerol, 0.1% casamino acids and 0.05% tyloxapol) with 0.02% of the indicated short chain fatty acid. After 2 days of exposure, RNA was isolated using the Direct-Zol RNA miniprep kit (Zymo Research) and cDNA was generated using Superscript IV reverse transcriptase (Invitrogen) with 200–300 ng of total RNA as a template. The transcription of *prpD*,*prpR*,*icl1* and *sigA* was measured with the primers included in Supplementary Table S1. The transcriptional levels of *prpD*,*prpR and icl1* were quantified using the delta-delta CT method using *sigA* as a housekeeping gene to normalize for input. Expression of the *prpR* wild-type complement in acetate was set to one in each case.

In the *ramB* knockdown experiments, wild-type H37Rv and the H37RvΔ*prpR* mutant strains transformed with the *ramB* knockdown vector were cultured in acetate or propionate media as describe above simultaneously with 100 ng ml^−1^ ATc where indicated to induce knockdown. RNA was collected after 48 h of media exposure and induction, and was quantified as described above.

## RESULTS

### Identification of a [4Fe4S] cluster in MtPrpR

To understand the transcriptional activation of MCC at the molecular level, we employed X-ray crystallography to characterize the structure and function of its transcriptional regulator MtPrpR. We first generated several recombinant protein expression plasmids of different constructs of MtPrpR designed based on the secondary structure and domain predictions (Supplementary Figure S2A). Each plasmid was tested for MtPrpR expression in *E. coli* strain BL21(DE3). Most expression constructs yielded protein that was either insoluble or aggregated during the purification procedure (Supplementary Figure S2B). However, when the first 80 residues that contained the N-terminal helix-turn-helix DNA-binding domain (residues 20–76) and its flanking loops were truncated, the resultant MtPrpR_81–486_ protein was both soluble and monodispersed at concentration exceeding 20 mg ml^−1^. Size exclusion chromatography (SEC) revealed the protein was a tetramer (Supplementary Figure S2C).

Recombinant MtPrpR_81–486_ was brown in color, and showed an absorbance peak at 410 nm in UV-visible spectrometry (Supplementary Figure S3A), matching the absorbance of [4Fe4S] cluster. We determined the extinction coefficient at 410 nm (ϵ410 nm) of the purified MtPrpR_81–486_ and it was ∼13 700 cm^−1^ M^−1^. Inductively coupled plasma mass spectrometry (ICP-MS) confirmed that the identity of the metal was iron and that each polypeptide chain contained approximately four irons (data not shown). Iron-sulfur clusters are typically oxygen labile and require stringent anaerobic conditions for the protein purification or cluster reconstitution. However, aerobically purified MtPrpR_81–486_ showed no apparent difference in solubility or UV-visible spectra compared to protein purified anaerobically. The EPR spectrum of untreated MtPrpR_81–486_ showed a weak signal at *g* = 2.015 (Supplementary Figure S3B), corresponding to the paramagnetic [3Fe4S]^+^ cluster. The concentration of this species was quantified, using a Cu(II)-EDTA standard, to be <2% of the total protein concentration. We did not detect other paramagnetic iron-sulfur cluster species including [4Fe4S]^+^ or [4Fe4S]^3+^, indicating the majority of the cluster in the protein should be the EPR-silent diamagnetic [4Fe4S]^2+^ form. Since oxidative degradation of a [4Fe4S]^2+^ cluster produces a [3Fe4S]^+^ cluster (50), a small portion of [3Fe4S]^+^ was expected, given that the protein was purified aerobically without cluster reconstitution. Intriguingly, the [4Fe4S]^2+^ cluster in MtPrpR_81–486_ appears to be resistant to oxidants, reductants or chelators (Supplementary Figure S3B). Treatment with 5-fold molar excess of ferricyanide ion did not increase the amount of [3Fe4S]^+^ species. Dithionite ion at a 10-fold molar excess did not reduce the EPR-silent [4Fe4S]^2+^ to the paramagnetic [4Fe4S]^+^ form, nor did it convert the paramagnetic [3Fe4S]^+^ to the EPR-silent [3Fe4S]^0^ form. A >20-fold molar excess of EDTA also failed to alter the EPR spectrum. The above results indicate that a physiologically relevant transition between the [4Fe4S] and the [3Fe4S] clusters is unlikely.

UV-visible spectra showed that the [4Fe4S] cluster of MtPrpR_81–486_ could tolerate 0.01% (∼3 mM) of H_2_O_2_ (Supplementary Figure S3C) or a 32-fold molar excess of nitric oxide (1.6 mM) (Supplementary Figure S3D) for hours without showing a decrease of the absorbance peak at 410 nm. The cluster could only be reduced by >30-fold molar excess of dithionite ion (1 mM) (Supplementary Figure S3E). This high level of redox stability is very unusual for an iron-sulfur protein. Therefore, MtPrpR is unlikely to be a redox sensor in the cell. We next assessed the importance of iron in culture media to the expression levels of the recombinant MtPrpR by expressing MtPrpR_81–486_ in M9-dextrose media supplemented with common transition metal (Fe, Co, Ni, Cu and Zn) ions. We could obtain soluble MtPrpR_81–486_ only when iron was added to the media (Supplementary Figure S3F).

### Crystal structure of MtPrpR_81-486_

We conducted crystallization trials on the above mentioned recombinant proteins, and only the stable MtPrpR_81–486_ containing the N-terminal His-tag produced protein crystals. An X-ray diffraction dataset was collected and processed at 2.7 Å resolution and phased using the single-wavelength anomalous diffraction (SAD) methods (51,52) with the selenomethionine-derived (Se-Met) protein crystals (Supplementary Table S2). Interestingly, the Se-Met protein crystallized in the P2_1_2_1_2_1_ space group with two axes of very similar lengths (a = 142.37 Å, b = 145.96 Å). However, the native protein crystallized in a similarly packed unit cell but in the higher symmetry space group, P4_1_2_1_2, with a = b = 144.87 Å. The asymmetric unit (ASU) of the P4_1_2_1_2 crystal contained two protein molecules and was equivalent to half of the ASU of the P2_1_2_1_2_1_ crystal. Nevertheless, the overall crystal unit cell volumes and the structures of both forms were nearly identical. Protein assembly analysis using the program PISA (45) showed that MtPrpR_81–486_ in the crystal was a homotetramer (Figure 1C), which was consistent with the SEC results (Supplementary Figure S2C). The four polypeptide chains organized in D_2_ symmetry. The shared surface area of the interface on each chain were around 2400 Å^2^ between Chains A and B; 1250 Å^2^ between Chains A and C; 340 Å^2^ between Chains A and D. Since the four chains in the Se-Met structure were virtually identical (Cα_RMSD < 0.3 Å), we focus on Chain A for subsequent structure depictions.

MtPrpR contains three domains (Figure 1B) aside from the N-terminal DNA-binding domain (DBD, residues 20–76) which was removed in the MtPrpR_81–486_ protein construct. A possibly disordered domain (designated D_II_, residues 81–152) connected the DBD to the third domain (designated D_III_, residues 153–324). The electron density for D_II_ was not visible in our crystal structure, but its presence in the crystallized protein was confirmed by protein mass spectrometry (Supplementary Figure S4). The D_III_ domain is composed of a nine-helix bundle and a three-stranded β-sheet (Figure 1D). A loop (residues 325–334) links the D_III_ to a GAF-like (cGMP phosphodiesterase/adenylyl cyclase/FhlA) (53) domain (residues 335–419).

MtPrpR also contains a long C-terminal region (residues 420–486) consisting primarily of loops plus several short secondary structure elements. The C-terminal region of Chain A interacts with the D_III_-GAF di-domain of Chain B in a head-to-tail manner (Figure 1C and D). The interaction is stabilized by an extended β-sheet formation between a short β-strand at the end of the C-terminal region and the three β-strands in the D_III_ domain of the neighboring chain.

The cubane-type [4Fe4S] cluster resides in the C-terminal region (Figure 1D, E) with three of the four irons interacting with Cys447, Cys450 and Cys455 in a ^447^C-X_2_-C-X_4_-C^455^ motif which appears to be conserved among all MtPrpR homologs (Supplementary Figure S5). Cys363 from the GAF-like domain of the same chain ligates the fourth iron. The loop harboring the C-X_2_-C-X_4_-C motif wraps around the [4Fe4S] cluster and buries the majority of it inside the MtPrpR_81–486_ tetramer, which we propose as the major contribution to the stability of the cluster when the protein is exposed to air.

A 3D structure similarity search of the MtPrpR_81–486_ structure against the Protein Data Bank (PDB) (46) showed that apart from the hits on the GAF-like domain, the only structure that showed significant similarity to MtPrpR_81–486_ was a *Deinococcus* radiation response regulator IrrE (22) (PDB code: 3DTI and related entries, Cα_RMSD 5.0 Å, Supplementary Figure S6A). There were only 240 residues within the D_III_-GAF region of MtPrpR_81–486_ that could be structurally aligned to IrrE, and the sequence identity of this region was only 14%. IrrE was reported as a metalloprotease with an HEXXH motif coordinating a Zn ion (22,23) (Supplementary Figure S6B). However, those residues are not conserved in MtPrpR.

### Identification of Coenzyme A in MtPrpR

**Figure 2.**
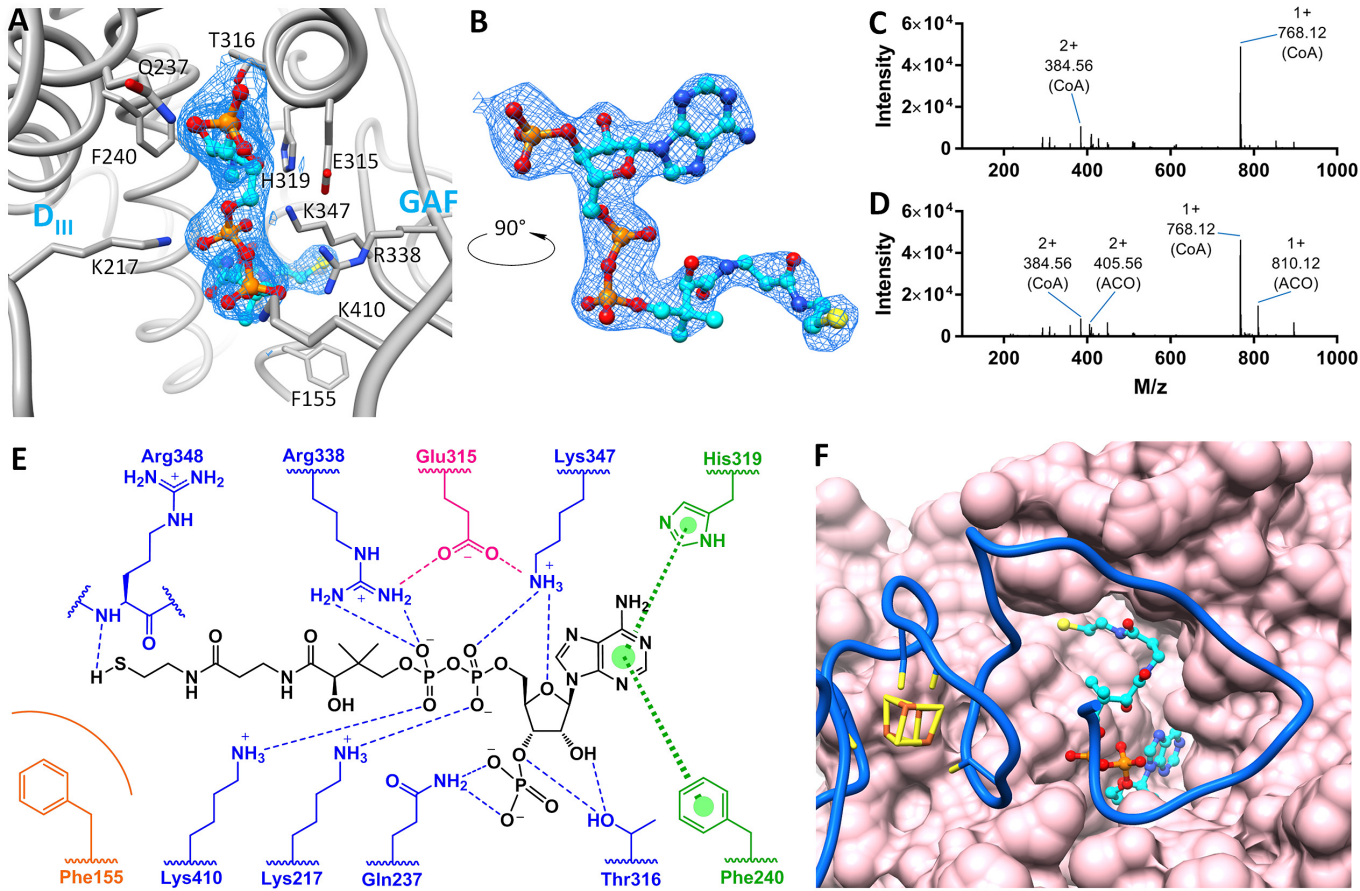
MtPrpR CoA binding cavity. (**A**) The CoA binding cavity in MtPrpR_81–486_; the protein chain is colored in gray. The binding cavity is formed by the D_III_ (left) and the GAF-like (right) domains. Key interacting residues are labeled. CoA is shown in stick and ball; the 2mFo-DFc map of CoA is contoured at 1 σ. (**B**) Electron density of CoA in A is displayed by a 90-degree rotation. (**C** and **D**) Mass spectra of CoA and its derivatives extracted from purified MtPrpR_81–486_ expressed in LB (C) or M9-dextrose-iron (D) media. M/z values and identities are labeled (ACO for acetyl-CoA). (**E**) CoA interacting residues. The π–π interactions are indicated by bold broken lines and colored in green; hydrogen bonds (also including electrostatic interactions) are indicated by dashed lines (blue for interactions between protein and CoA; magenta for interactions to stabilize the side-chain rotamer conformation); orange arc line indicates van der Waals interactions. (**F**) CoA-binding cavity of Chain B (pink for protein surface, cyan for CoA) covered by the neighboring Chain A (blue).

After building and refining the protein atoms and the [4Fe4S] clusters into the electron density map, we observed a relatively large segment of electron density in each polypeptide chain that could not be accounted for by protein or chemicals in the purification buffer or crystallization condition. This electron density was nearly identical in shape in each of the four subunits, and was located in the cavity between the D_III_ and GAF-like domains in both the mFo-DFc and 2mFo-DFc difference electron density maps (Figure 2A and B). This indicated that a ligand had been cocrystallized with the protein. To identify the ligand, MtPrpR_81–486_ was denatured, and the ligand was enriched by ion exchange chromatography followed by liquid chromatography-high resolution mass spectrometry (LC-HRMS). The mass to charge (*m/z*) values showed that the majority of the ligand was Coenzyme A ([M+H]^+^*m/z* of 768.12) from protein expressed in LB media (Figure 2C). When the protein was expressed in M9-dextrose-iron media, acetyl-CoA ([M+H]^+^*m/z* of 810.12) was also detected in addition to CoA (Figure 2D). The ratio of acetyl-CoA to CoA was about 1:8 according to the HPLC UV chromatogram (data not shown). However, we did not observe well-defined electron density for the acetyl group, although there was room for it in the binding cavity. Therefore, the predominant CoA was built into all protein chains and the CoA molecules fit into the electron density. The refined mean B-factors for the CoA was 69.43 Å^2^, consistent with the mean B-factor of 67.28 Å^2^ for the protein. As mentioned earlier, the D_III_ and GAF-like domains of MtPrpR are structurally similar to *Deinococcus* IrrE protein. Notably, IrrE also possesses a deep cavity which is the counterpart of the CoA-binding cavity in MtPrpR (Supplementary Figure S6A). Although vacant in all reported IrrE structures, the cavity may function to accommodate radiation-sensing ligands, given that IrrE is a radiation response protein.

Although the CoA-binding pocket was composed in part by the GAF-like domain of MtPrpR, the binding mode was quite different from those found in typical GAF domain structures. The canonical GAF domain and the closely related PAS (Per-Arnt-Sim) domain have been reported to bind to a variety of signaling molecules (53–57) on the concave face of the central β-sheet of the GAF domain (Supplementary Figure S7A and B). However, the CoA-binding cavity in MtPrpR was cooperatively formed between the convex face of the central β-sheet of the GAF-like domain and the helix bundle of the D_III_ domain (Figure 2A; Supplementary Figure S7A and B). The adenine group was sandwiched between Phe240 and His319 and was held in place by π-π interactions. The rest of CoA forms a host of hydrogen bonds and electrostatic interactions with the protein, contributed by the side chains or backbone atoms of seven amino acids in the D_III_ and GAF-like domains (Figure 2A and E). The diphosphate group in particular was surrounded by four basic residues, Lys217, Lys347, Lys410 and Arg338. Phe155, located at the bottom of CoA-binding cavity, contacted the thiol group of CoA through van der Waals interactions. The C-terminal loop region of neighboring Chain B forms a hinged triangular lid that covers the CoA-binding cavity of Chain A (Figure 2F). The hinge region harbors the ^447^C-X_2_-C-X_4_-C^455^ motif where the [4Fe4S] cluster binds. The cluster appears to stiffen the hinge region, as indicated by the lower local B-factors (Supplementary Figure S7C and D), making it difficult for the lid to open, hence locking CoA inside the cavity. Common ligand stripping procedures such as dialysis failed to dissociate CoA from the protein. Based on the structure, the integrity of the iron-sulfur cluster appears to be pivotal for protein tetramerization and the retention of CoA in the cavity.

### The [4Fe4S] cluster is critical for CoA binding and transcription activation

**Figure 3.**
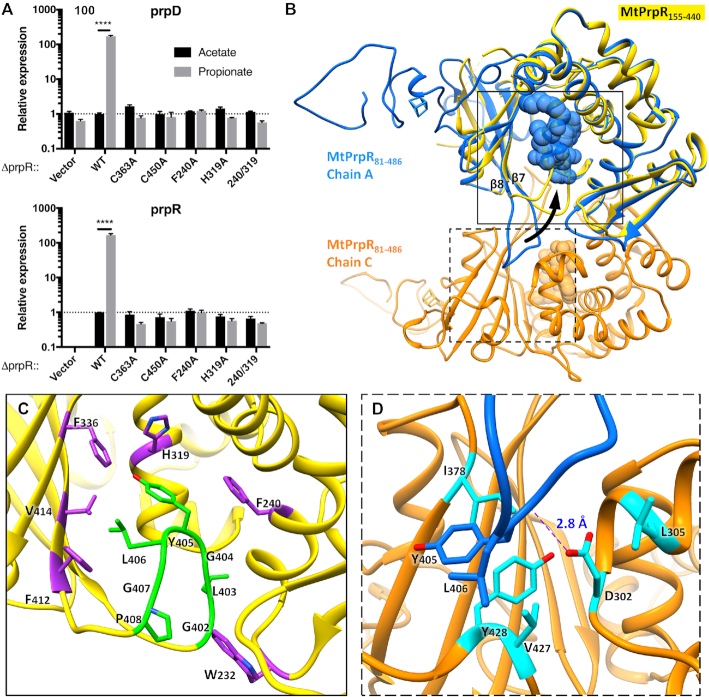
Effect of [4Fe4S] cluster upon MtPrpR. (**A**) Transcription levels of *prp*D (*N* = 3) and *prpR* (*N* = 3) in MtPrpR variants under acetate or propionate conditions. Values presented as mean ± SD (Data were analyzed with two-way ANOVA followed by Sidak's test, **** *P* < 0.0001). (**B**) Conformational change of MtPrpR in the presence (blue and orange for the two neighboring chains of MtPrpR_81–486_) or absence (yellow for MtPrpR_155–440_) of the [4Fe4S] cluster binding domain. The arrow indicates the open and closed conformations of the loop; Regions within the solid and dashed line boxes are zoomed in with detailed interactions shown in (**C**) and (**D**).

To determine if the [4Fe4S] cluster in the structure is important for transcriptional regulation, we picked two iron-ligating cysteine residues, Cys363 in the GAF-like domain and Cys450 in the C-terminal ^447^C-X_2_-C-X_4_-C^455^ motif and mutated them individually into alanine (C363A and C450A) using a *prpR*-containing shuttle vector (17). The wild-type *prpR*, each mutant and an empty vector were individually integrated into the chromosome of *prpR*-deleted *Mtb* laboratory strain H37Rv (H37RvΔ*prpR*) (8). The impact on transcription levels were compared between propionate and acetate carbon sources because both molecules are nonfermentable while only propionate elevates *prp* operon transcription. Upregulation of the *prp* operon, represented by *prpD* or *prpR* in response to propionate were completely abolished in the Δ*prpR::C363A*, Δ*prpR::C450A*, and Δ*prpR::vector* strains (Figure 3A).

We next sought to visualize the iron-sulfur free form of the protein. Several methods have been attempted to obtain iron-free protein. Overexpressing MtPrpR_81–486_ in M9-dextrose media without supplementing iron failed to produce soluble protein. Incubating purified MtPrpR_81–486_ in the SEC buffer containing 10 mM EDTA also failed to strip iron from the protein or alter its tetrameric state as shown by SEC (Supplementary Figure S2D). We then truncated a large portion of the C-terminal region (residues 441–486) containing the ^447^C-X_2_-C-X_4_-C^455^ iron-sulfur cluster-binding motif. In addition, residues 81–154, which were not visible in the MtPrpR_81–486_ structure due to flexibility, were also removed from the expression construct. The resultant *prpR_155–440_* expression construct produced soluble protein with no color and the protein was a monomer in solution as shown by SEC (Supplementary Figure S2E). The crystal structure of MtPrpR_155–440_ showed that the protein retained the D_III_-GAF core structure of MtPrpR_81–486_ and, as expected, had no bound [4Fe4S] cluster. However, CoA was also absent in the structure, even though 1 mM CoA was incubated with the protein during crystallization.

The iron-free structure revealed a dramatic conformational change of the protein (Figure 3B). The loop between β7 and β8 of the GAF-like domain flipped back nearly 180 degrees to be oriented towards the CoA-binding cavity and blocked the cavity entrance. A hydrophobic fragment of the loop with the amino acid sequence of ^402^GLGYLGP^408^ inserted into the cavity, interacting with Phe240, Phe336, Phe412, Trp232, His319 and Val414, all within van der Waals distances to the loop (Figure 3C). Gly404, Tyr405 and Leu406 at the tip of the loop occupy the space where the 3′-phosphoadenosine diphosphate moiety of CoA resided in the CoA-bound form of the protein (Figure 3B and C). While in the tetrameric MtPrpR_81–486_ structure, the loop was held in an open conformation mainly through hydrophobic interactions with the neighboring chains. For example, the loop in Chain A was surrounded by Leu305, Ile378, Val427 and Tyr428 in the neighboring Chain C, all within a distance of 5 Å (Figure 3D). In addition, Asp302 in Chain C hydrogen bonded (2.8 Å) to the backbone nitrogen of Tyr405. These interactions hold the hydrophobic loop in an open conformation allowing for CoA binding. Taken together, the iron-sulfur cluster determines the ability of MtPrpR to bind CoA by regulating the conformation of this hydrophobic loop.

### CoA derivatives control the MCC pathway activation

The π–π interactions between the CoA adenine group and the residues Phe240 and His319 are likely to play critical roles during CoA binding. These two residues are invariant in most MtPrpR homologs (Supplementary Figure S5). To test the importance of these interactions, we complemented the H37RvΔ*prpR* strain with *prpR_F240A, prpR_H319A* single mutations or *prpR_F240A/H319A* double mutation. None of the resultant strains could upregulate *prpD* or *prpR* in response to propionate (Figure 3A).

There is good agreement between our studies and the literature (8,15) that show *prpR* activates the transcription of the *prp* operon during propionate or cholesterol carbon utilization, where propionyl-CoA is the common metabolite. Therefore, we speculated that propionyl-CoA, rather than the observed CoA or acetyl-CoA, was the co-activator of the *prp* operon. We tried to crystallize propionyl-CoA bound MtPrpR_81–486_ by mixing the protein with different concentrations of propionyl-CoA, or by purifying MtPrpR_81–486_ expressed in M9-propionate media. Nonetheless, the desired propionyl group was not discernible in the electron density map. This was likely due to the closed conformation of the CoA-binding pocket as described above.

**Figure 4.**
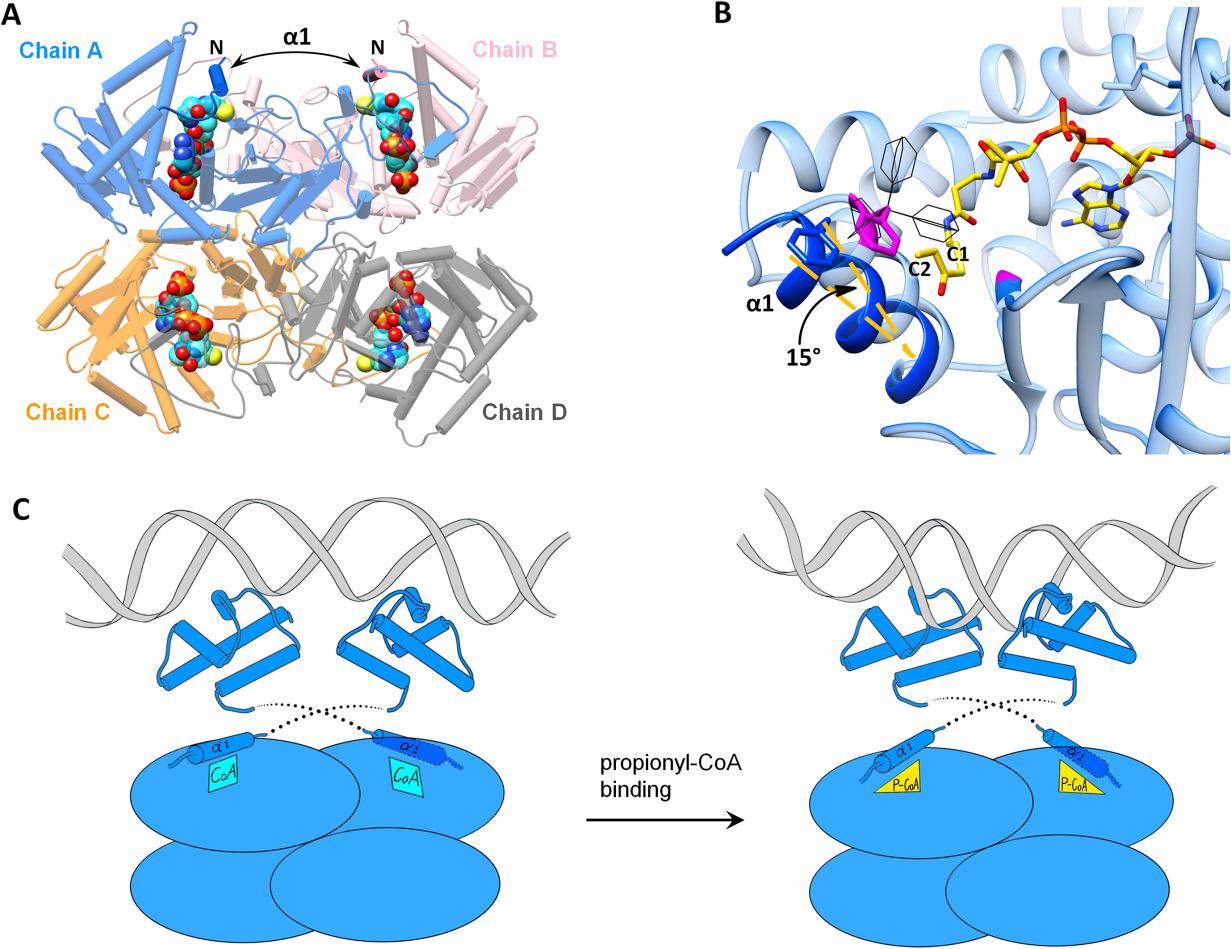
Proposed model of MtPrpR conformational change and transcriptional activation. (**A**) Location of helix α1 in Chains A and B in MtPrpR_81–486_ tetramer. The N-termini of the visible portion of the protein are shown. (**B**) Model of propionyl-CoA bound in the CoA-binding cavity of Chain A (light blue). Atoms within 4 Å to C1 and C2 of the propionyl group are colored in purple. Rotamers of Phe155 are shown as black wires. A minimum of 15° movement of α1 (dark blue for the new position) is required to overcome the clashes with propionyl-CoA. (**C**) Schematic of MtPrpR-mediated transcriptional regulation. Binding of propionyl-CoA is proposed to induce a conformational change of MtPrpR via helix α1, which may alter the distances between the adjacent HTH domains and bend the recognition DNA, leading to gene activation.

We computationally modeled propionyl-CoA into the MtPrpR_81–486_ structure by treating the protein as a rigid body. This led to steric clashes between the propionyl group and Phe155 which is located in the first visible α-helix (α1) in the crystal structure (Figure 4A and B). These clashes could not be overcome by simply changing the rotamer conformation of Phe155. Instead, a roughly 15-degree movement of α1 is required for the protein to accommodate propionyl-CoA (Figure 4B). This implies that Phe155 directly participates in the ligand selection and that the CoA-bound MtPrpR was transcriptionally inactive while the propionyl-CoA-bound form should undergo a conformational change and becomes active. The active form of MtPrpR may alter the recognition DNA conformation, leading to the transcriptional activation of the *prp* operon (Figure 4C).

**Figure 5.**
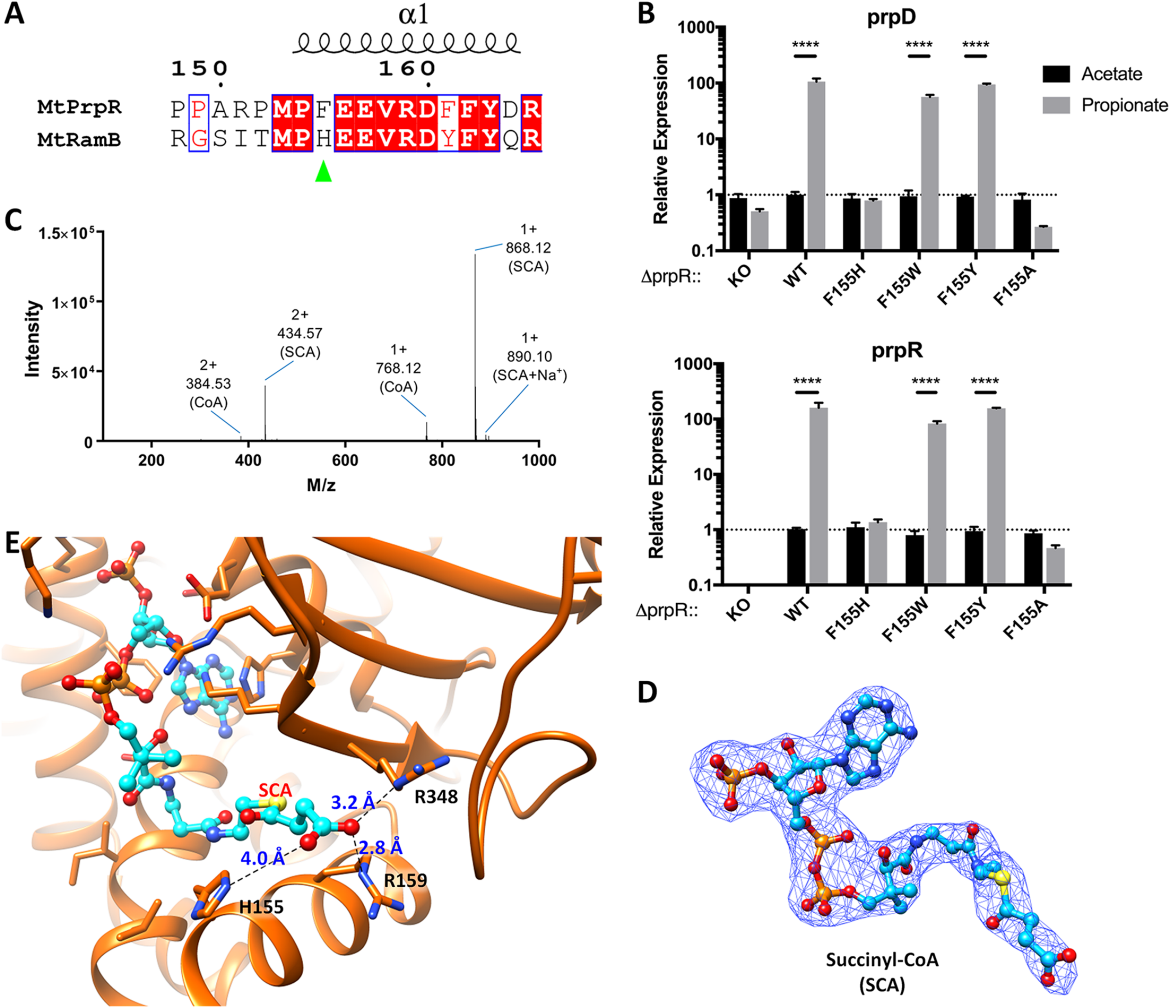
The polymorphism of residue 155 (number as in MtPrpR) in the MtPrpR homologs controls the ligand selectivity. (**A**) Sequence alignment of the helix α1 between MtPrpR and MtRamB, highlighting Phe155 in MtPrpR and its counterpart His143 in MtRamB. (**B**) Transcription levels of *prp*D (*N* = 3) and *prpR* (*N* = 3) in MtPrpR_F155 variants under acetate or propionate conditions. Values presented as mean ± SD (Data were analyzed with two-way ANOVA followed by Sidak's test, **** *P* < 0.0001). (**C**) Mass spectrum of the ligands. *M/z* and ligand identities are labeled (SCA for succinyl-CoA; SCA+Na^+^ for the sodium adduct form of SCA). (**D**) The 2mFo-DFc electron density of succinyl-CoA bound by MtPrpR_81–486__F155H mutant, electron density contoured at 1 σ. (**E**) The succinyl-CoA binding environment. The dashed lines indicate the interactions between the protein and the succinyl moiety.

Through a structure-guided sequence comparison, we found that Phe155 in MtPrpR was replaced by a histidine in its paralog MtRamB (Phe155→His143, Figure 5A), while other CoA-contacting residues were identical. Recall that MtRamB has been shown as a transcriptional repressor of *icl1* in dextrose containing media (24). We hypothesized that the Phe→His substitution would allow MtRamB to bind to a different CoA derivative, and that F155H mutation of MtPrpR would affect its ability to respond to propionyl-CoA. We therefore integrated a *prpR_F155H* mutant allele into the H37RvΔ*prpR* genome. The Δ*prpR::F155H* retained little or no activity as measured by the strongly attenuated upregulation of both *prpD* and *prpR* in response to propionate in two experimental runs (Figure 5B and Supplementary Figure S8A). Mutating Phe155 into alanine, which has an even smaller side chain, completely abrogated the upregulation of *prpD* and *prpR*, while mutating Phe155 into the bulky aromatic residues tryptophan or tyrosine restored the upregulation to the wild-type level (Figure 5B). Notably, these bulky aromatic residues naturally exist in MtPrpR homologs activating either the MCC (i.e. Ajs_1637 protein of *Acidovorax sp. JS42* strain harbors a Tyr (58)) or the methylmalonyl pathway (i.e. the PccR protein of *Rhodobacter sphaeroides* harbors a Trp (26)) (Supplementary Figure S5). Both pathways are used by bacteria to assimilate propionyl-CoA. Therefore, it stands to reason that propionyl-CoA clashes with those aromatic residues and induces similar conformational changes in the proteins for transcriptional activation.

### An MtRamB-like mutant of MtPrpR binds to succinyl-CoA

We were particularly interested in the identity of the ligand recognized by MtRamB. However, the poor solubility of the full-length MtRamB and the aggregation of MtRamB_69–474_ (truncated to corresponding positions of MtPrpR_81–486_) obstructed a direct study of MtRamB. We instead used MtPrpR_81–486__F155H as a mimic based on the fact that the other CoA-binding residues in both proteins are identical. MtPrpR_81–486__F155H remained a tetramer in solution as shown by SEC (Supplementary Figure S2C) and crystallized in the same condition as the wild-type protein. The overall structure of the polypeptide chain superimposed perfectly to the CoA-bound wild-type protein, including the helix α1 where residue 155 is located (Cα_RMSD = 0.314 Å, Supplementary Figure S8B).

The 2mFo-DFc and mFo-DFc electron density maps showed that MtPrpR_81–486__F155H also bound to a ligand whose electron density matched with CoA in general except that the ligand was longer than CoA (Supplementary Figure S8C). This indicated that a fatty acyl-CoA derivative was bound by the mutant protein. The ligand was extracted and identified using LC-HRMS and a major [M+H]^+^ peak with an *m/z* of 868.12 was detected (Figure 5C) in addition to CoA. The amount of CoA was less than a quarter of the new compound according to the UV chromatogram (data not shown). This mass corresponds to both epimers of methylmalonyl-CoA as well as succinyl-CoA, which share an identical chemical formula. Attempting to build (R)- or (S)- methylmalonyl-CoA into the electron density resulted in a strong negative electron density peak (above 4 σ) around the methyl branch in the mFo-DFc map (Supplementary Figure S8D). The methyl branch also caused steric clashes with the adjacent residues His155 and Arg159, while succinyl-CoA fit the electron density without steric overlap (Figure 5D). The carboxyl group of the succinyl moiety interacts with His155 (∼4 Å), Arg348, and Arg159 (∼2.8–3.2 Å) (Figure 5E).

Since the MtPrpR_81–486__F155H mutant was generated to make the ligand-binding residues identical to those of MtRamB, the observation of succinyl-CoA bound by this mutant indicates that succinyl-CoA is likely also bound by MtRamB.

### MtPrpR and MtRamB cross-regulate the transcription of *icl1* but not *prp* operon

**Figure 6.**
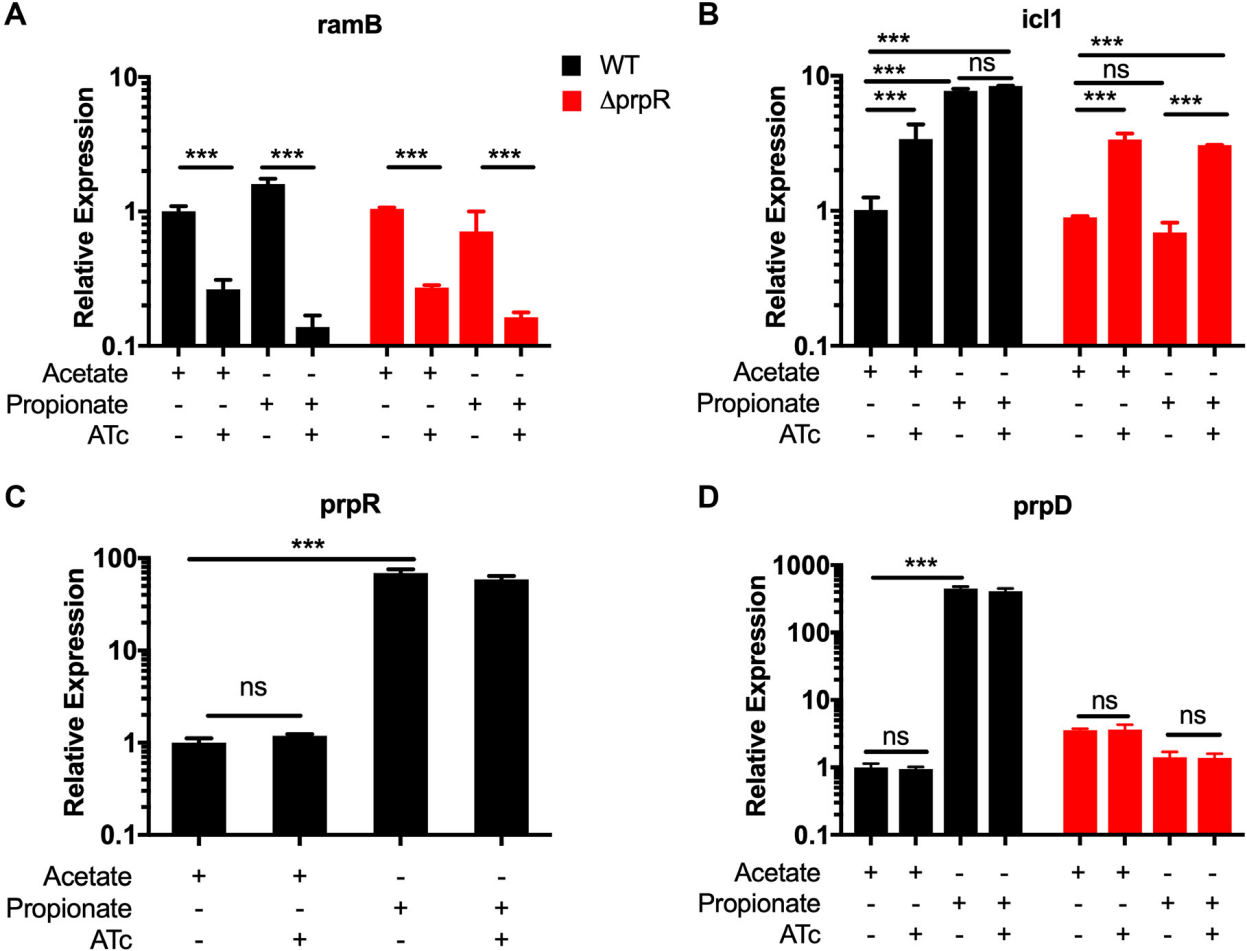
Transcriptional regulation by MtPrpR and MtRamB on *prp* operon and *icl1*. (**A**) Transcription levels of *ramB* in *Mtb* H37Rv or Δ*prpR* strains using acetate or propionate carbon sources with or without CRISPRi induction by anhydrous tetracycline (ATc). (**B**–**D**) Transcription levels of *icl1*,*prpR and prpD*, respectively, under the same treatment as in A. The black and red bars are WT H37Rv and H37RvΔ*prpR*, respectively, transformed with an ATc inducible vector. *N* = 2 for each strain, treatment and carbon source combination. Data were analyzed with two-way ANOVA followed by Tukey's test. *** *P* < 0.0002, ns: not significant.

Given that *Mtb* has two homologous regulators controlling related pathways, crosstalk between *prpR* and *ramB* is very likely. To investigate this, we incorporated a tunable *ramB* knockdown into both wild-type and the *prpR*-deleted backgrounds using a CRISPR interference (CRISPRi) system optimized for *Mtb* (27). The knockdown of *ramB* was confirmed as measured by a depletion of 70–90% of its transcription after anhydrous tetracycline (ATc) induction (Figure 6A).

Our studies showed that *icl1* transcription was repressed by MtRamB and activated by MtPrpR. Specifically, by depleting *ramB* through ATc induction, *icl1* transcription increased by at least 2-fold in the wild-type strain during acetate utilization and the Δ*prpR* strain during both acetate and propionate utilization (Figure 6B). However, when propionate is utilized by the wild-type strain, MtPrpR upregulated *icl1* transcription by approximately 7-fold compared to acetate utilization, and depleting *ramB* did not further increase *icl1* transcription (Figure 6B). The above results reveal a dominant role of MtPrpR during propionate metabolism. The upregulation of *icl1* during propionate metabolism can be explained by the protein's MCL activity (12,13) in the *Mtb* MCC.

Unlike *icl1, prpD* and *prpR* exclusively engage in MCC, and thus were only upregulated by MtPrpR in response to propionate utlization (Figure 6C and D). Depleting *ramB* had no noticeable effect on the transcription level of *prpD* or *prpR* under either carbon source.

## DISCUSSION

*Mtb*’s dependence on lipid catabolism (2), especially cholesterol (3), is critical for establishing a chronic infection, as is its reliance on the MCC to assimilate propionyl-CoA and detoxify its metabolic intermediates (11,12,20). The transcription factor MtPrpR determines the on and off states of the MCC (8,14,15). However, the triggering molecule that activates the MCC was not known, and consequently, the biochemical mechanism of the regulation has remained a mystery. Our study showed that MtPrpR directly binds to CoA or CoA derivatives, and that the binding is under the control of an iron-sulfur cluster which is located at the C-terminal region of the protein in close proximity to the CoA-binding cavity of a neighboring chain.

Based on the crystal structures and our model, it appears that MtPrpR can adopt inactive and active conformations by binding to different CoA derivatives. Specifically, the CoA- and acetyl-CoA-bound forms are transcriptionally inactive, while the propionyl-CoA-bound MtPrpR is likely to undergo a conformational change and becomes active. The MtPrpR_F155H mutant, on the other hand, was shown to bind to succinyl-CoA and was locked in a transcriptionally inactive conformation regardless of the carbon sources. Since MtPrpR_F155H was generated to harbor a CoA-binding pocket mimicking that of MtRamB, we suggest that MtRamB also binds to succinyl-CoA and is locked in a transcriptionally inactive conformation, which may explain the repressive role of MtRamB.

**Figure 7.**
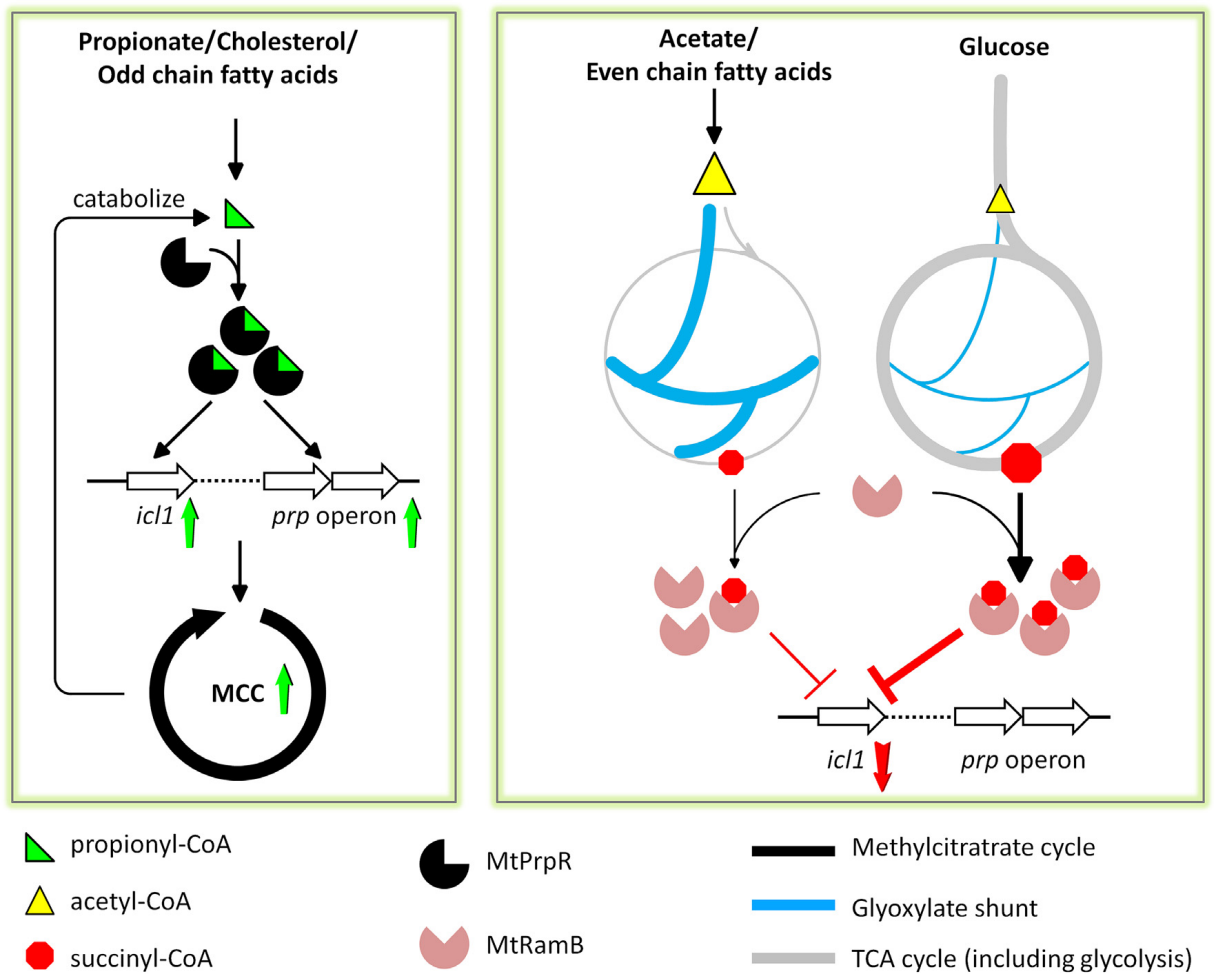
Schematic of MtPrpR/MtRamB regulation mediated by short-chain fatty acyl-CoAs. Left panel: MtPrpR-mediated transcriptional activation via propionyl-CoA binding. The upregulation of the *prp* operon and *icl1* leads to a robust MCC to efficiently assimilate and detoxify propionyl-CoA. Right panel: MtRamB-mediated transcriptional repression via succinyl-CoA binding. Succinyl-CoA can be produced at different levels depending on the carbon sources and the metabolic pathways including the glyoxylate shunt and the TCA cycle. Binding to succinyl-CoA by MtRamB leads to the transcriptional repression of *icl1* but not the *prp* operon.

Combining the structural and genetic information, we propose a regulatory model for the MCC and the glyoxylate shunt that is based on the binding of the corresponding CoA derivatives to MtPrpR and MtRamB (Figure 7). Under propionyl-CoA-generating conditions, such as cholesterol and odd-chain fatty acids, MtPrpR is switched to an active conformation by binding to propionyl-CoA and robustly upregulates the MCC genes to catabolize and detoxify propionyl-CoA. When acetyl-CoA but not propionyl-CoA is generated, such as non-fermentable carbon sources acetate and even-chain fatty acids and fermentable carbon source dextrose, the glyoxylate shunt and TCA cycle will be employed to assimilate acetyl-CoA. Catabolism of acetyl-CoA via the TCA cycle produces succinyl-CoA (see below) which will be bound by MtRamB for *icl1* transcriptional repression. It has been shown that dextrose could induce a strong transcriptional repression of *icl1* mediated by MtRamB (24). This indicates that dextrose degradation relies heavily on the TCA cycle where high concentration of succinyl-CoA can be generated. On the other hand, acetyl-CoA produced from acetate and fatty acids are mainly routed into the glyoxylate shunt and therefore the succinyl-CoA level will be lower, resulting in a weaker repression of *icl1*.

Previously, *Mtb* was shown to operate a variant TCA cycle which employs α-ketoglutarate (KG) decarboxylase and succinic semialdehyde dehydrogenase instead of KG dehydrogenase (KDH) and succinyl-CoA synthetase in the traditional TCA cycle (59). This finding led to the argument that succinyl-CoA was not an intermediate of *Mtb* TCA cycle. However, it has been later demonstrated that *Mtb* synthesizes succinyl-CoA from KG using both KDH (60,61) and KG:ferrodoxin oxidoreductase (KOR) (61). A high level of succinyl-CoA produced in the TCA cycle allows *Mtb* to temporarily shut down glyoxylate shunt mediated by MtRamB. Furthermore, it was recently reported the detection of Icl1 lysine succinylation when *Mtb* was cultured in a rich medium containing dextrose (62). The succinylation decreased the enzymatic activity of Icl1 (62,63). Taken together, it appears that an increased level of succinyl-CoA reduces the concentration of active icl1 at both the mRNA and the protein levels.

A sequence-based classification showed that MtPrpR homologs form a distinct clade of prokaryotic transcription factors that regulate acyl-CoA assimilation pathways including MCC, glyoxylate shunt, methylmalonyl pathway, ethylmalonyl-CoA pathway and several unresolved pathways (26). This clade all contain a C-X_2_-C-X_4_-C motif and very likely bind to [4Fe4S] cluster. We now refer to this clade as the iron-sulfur-dependent acyl-CoA regulators (IsaR) in order to distinguish them from the other non-iron-sulfur regulators that modulate acyl-CoA assimilation pathways (58).

A highly stable [4Fe4S] cluster is rare, but was previously reported in the *E. coli* endonuclease III (64,65) whose cluster was later shown to be activated upon DNA binding so as to sense DNA lesions (66). However, as a dedicated central carbon metabolic regulator, MtPrpR seems not engaged in measuring DNA integrity. There are several lines of evidence that indicate the role of [4Fe4S] cluster in MtPrpR is to sense iron availability in the cell and modulate *prp* operon transcription. Although [4Fe4S] and [2Fe2S] clusters have been reported to serve as iron sensors in IscR, RirA and IRP1 for iron acquisition and iron homeostasis (67), there has not been a case before where these iron sensors directly regulate the transcription of central carbon metabolism (CCM). We demonstrated that when [4Fe4S] cluster binding was disabled, MtPrpR could not bind to CoA derivatives or activate *prp* operon transcription. Recent studies on *Mtb* transcriptome (68) also showed that during iron-starvation the reduction of *prp* operon transcription was the most significant among all *Mtb* genes. Moreover, *Mtb* becomes non-replicating under iron-starvation (68). Therefore, lowering CCM level may be a strategy for *Mtb* to survive iron-restriction that is initiated by host immune system, and MtPrpR is likely a member of the iron-sensing network.

Previously, mutations in *prpR*, which resulted in a deficient MCC, have been found prevalent in clinically isolated drug resistant *Mtb* (17). The mutation sites span almost all over the MtPrpR protein. Interestingly, except for K217E, none of the mutations belong to CoA- or the iron-sulfur cluster-binding residues. Mutations flanking Phe155 were also observed, which include M153T and E156G, but Phe155 was invariable, as expected based on the functional importance of the residue in determining which CoA analogs can bind. So far, we are not able to rationalize the mode of action of these drug-resistant mutations from the structure.

Taken all together, our structure of the MtPrpR represents a prototype of the IsaR family of transcription factors. Moreover, despite the sequence dissimilarity, the protein folding of MtPrpR could be repurposed and widely used by different organisms to harness a variety of signaling pathways.

## DATA AVAILABILITY

Atomic coordinates and structure factors for the reported crystal structures have been deposited with the Protein Data Bank under accession numbers: 6CZ6, 6CYY, 6CYJ, 6D2S.

## Supplementary Material

gkz724_Supplemental_File

## Notes

Present address: Yu-Shan Cheng, National Center for Advancing Translational Sciences, National Institutes of Health, 9800 Medical Center Drive, Bethesda, MD 20892, USA.
