# Supplementary material for: Structural and functional insight into the Mycobacterium tuberculosis protein PrpR reveals a novel type of transcription factor
Source: Nucleic Acids Res. 2019 Aug 26;47(18):9934–49. doi: 10.1093/nar/gkz724 (PMC6765138; doi:10.1093/nar/gkz724)
Supplement: gkz724_Supplemental_File [file gkz724_supplemental_file.pdf]

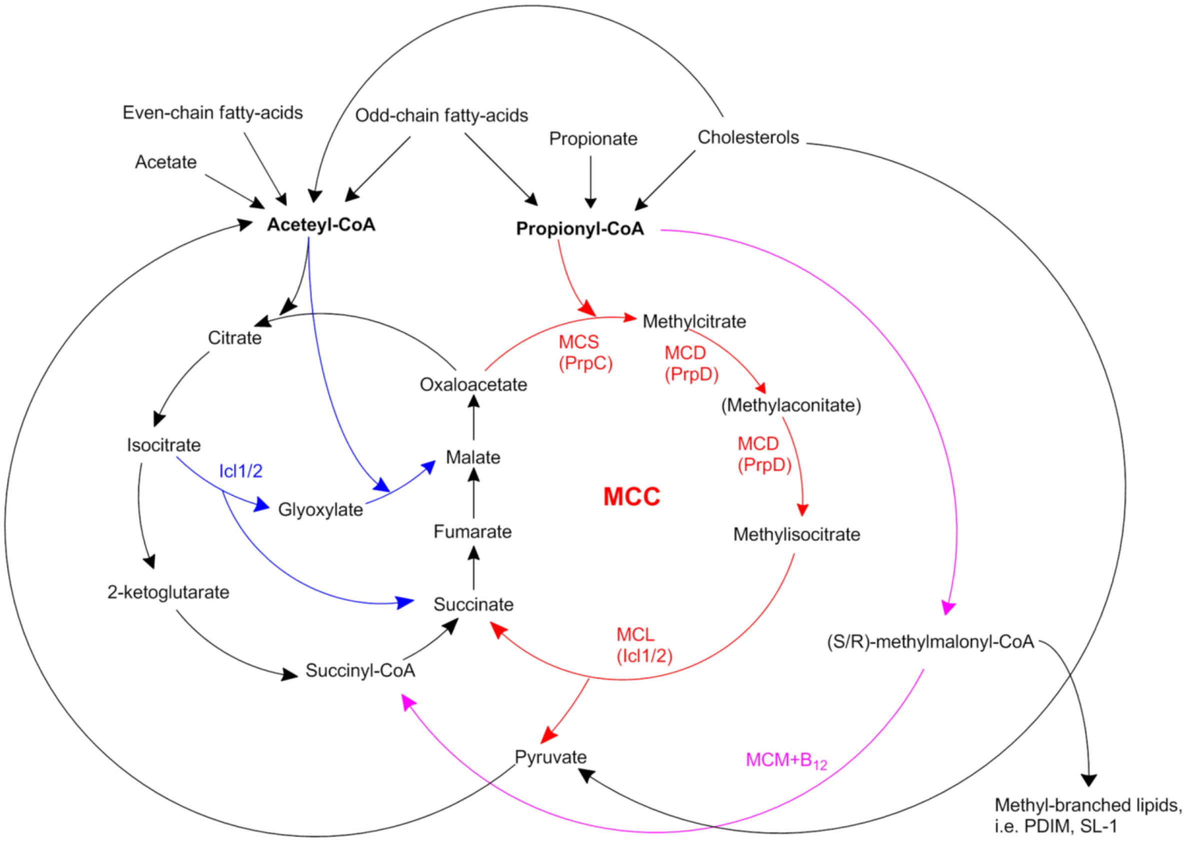

Supplementary Figure S1

A truncated central carbon metabolism in *Mtb* catabolizing propionyl-CoA and acetyl-CoA. Methylcitrate cycle (MCC) is colored red; glyoxylate shunt is colored blue; methylmalonyl pathway is colored magenta. Abbreviations: MCS, methylcitrate synthase; MCD, methylcitrate dehydratase; MCL, methylisocitrate lyase; MCM, methylmalonyl-CoA mutase; PDIM, phthiocerol dimycocerosate; SL-1, sulfolipid-1.

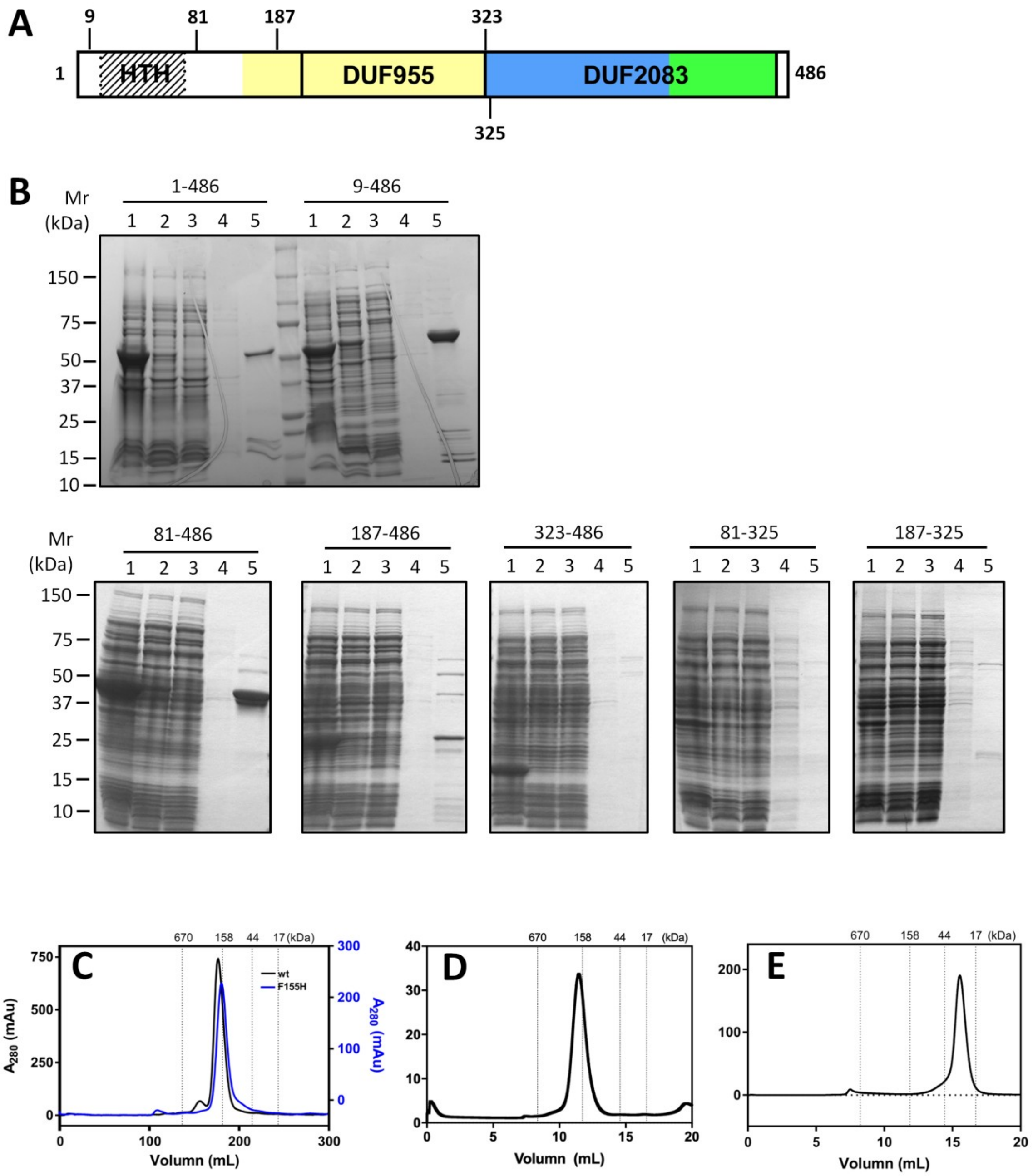

Supplementary Figure S2

(A) Truncations of MtPrpR. Numbers indicate the N- (above) and C-termini (below) of the constructs. Protein domains are illustrated as in Figure 1B. Black boxes show the original domain annotation and color fills are the domains observed in the crystal structure. **(B)** Affinity purification of each construct in **A**. Numbers above each lane indicates: 1. lysate, 2. supernatant, 3. flow-through, 4. wash, 5. elution). **(C), (D) and (E)** Size exclusion chromatography of different forms of MtPrpR: MtPrpR$_{81-486}$_WT and F155H (**C**), MtPrpR$_{81-486}$_WT incubating with 10 mM EDTA for 36 hr (**D**) MtPrpR155-440 (**E**).

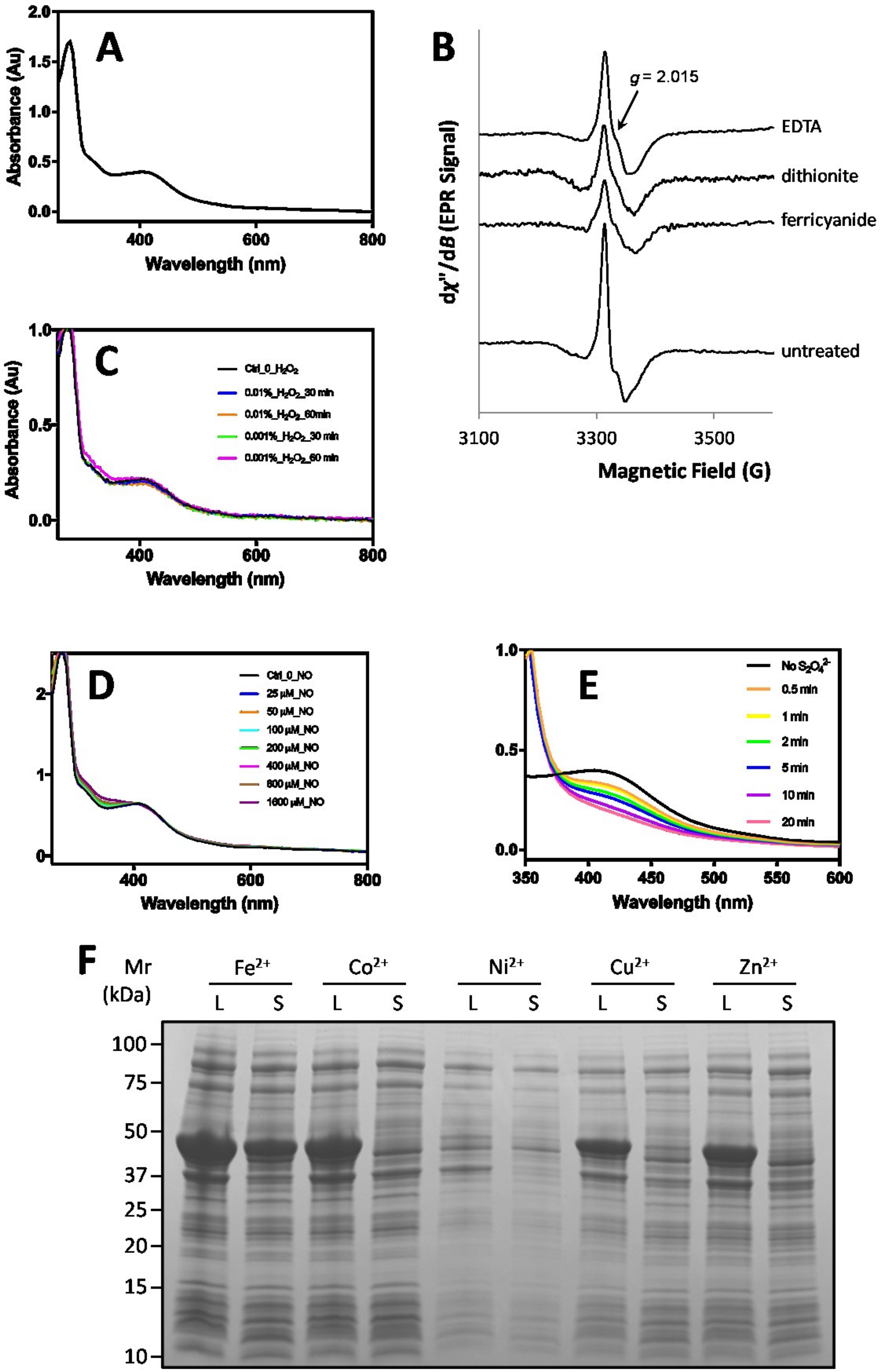

A
Absorbance (Au)
Wavelength (nm)
B
g = 2.015
EDTA
dithionite
ferricyanide
untreated
dχ''/dB (EPR Signal)
Magnetic Field (G)
C
Ctrl_0_H2O2
0.01%_H2O2_30 min
0.01%_H2O2_60min
0.001%_H2O2_30 min
0.001%_H2O2_60 min
D
Ctrl_0_NO
25 μM_NO
50 μM_NO
100 μM_NO
200 μM_NO
400 μM_NO
800 μM_NO
1600 μM_NO
E
No S2O4 2-
0.5 min
1 min
2 min
5 min
10 min
20 min
F
Mr
(kDa)
Fe2+
Co2+
Ni2+
Cu2+
Zn2+
L
S

Supplementary Figure S3

(A) UV-visible spectrophotometry of 30 µM $MtPrpR_{81\text{-}486}$. **(B)** EPR spectra of $MtPrpR_{81\text{-}486}$ at 250 µM and under different treatments. Spectra were recorded at 10 K. The microwave frequency was 9.38 GHz. The field modulation was 5 G at 100KHz and the microwave power was 0.2 mW. **(C)** Effect of indicated concentrations and incubation time of H_2O_2 upon 20 µM $MtPrpR_{81\text{-}486}$. **(D)** Effect of indicated concentrations of nitric oxide upon 50 µM $MtPrpR_{81\text{-}486}$. **(E)** Effect of 1 mM sodium dithionite upon 30 µM $MtPrpR_{81\text{-}48}$ with indicated incubation time. **(F)** Several common transition metals were tested to in addition to iron during $MtPrpR_{81\text{-}486}$ expression. Only iron could yield soluble protein (L, lysate; S, supernatant).

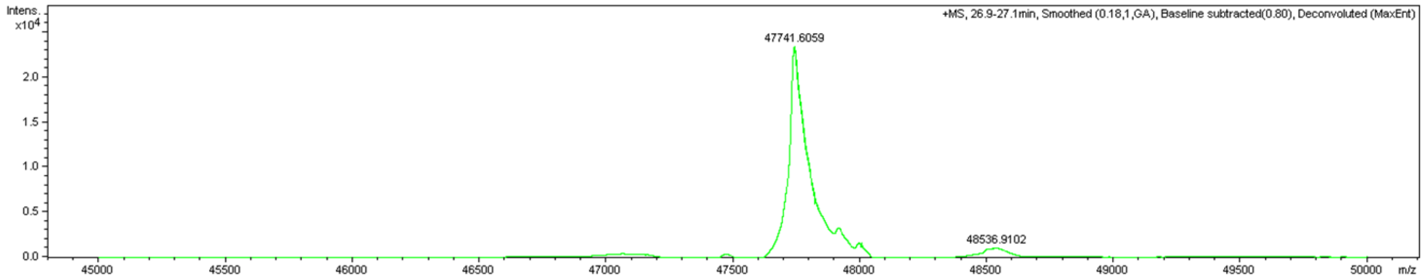

Supplementary Figure S4

Mass spectrometry of the His-tagged $MtPrpR_{81\text{-}486}$ crystals showed a deconvoluted peak with *m/z* of 47,741, indicating that no proteolysis had occurred.

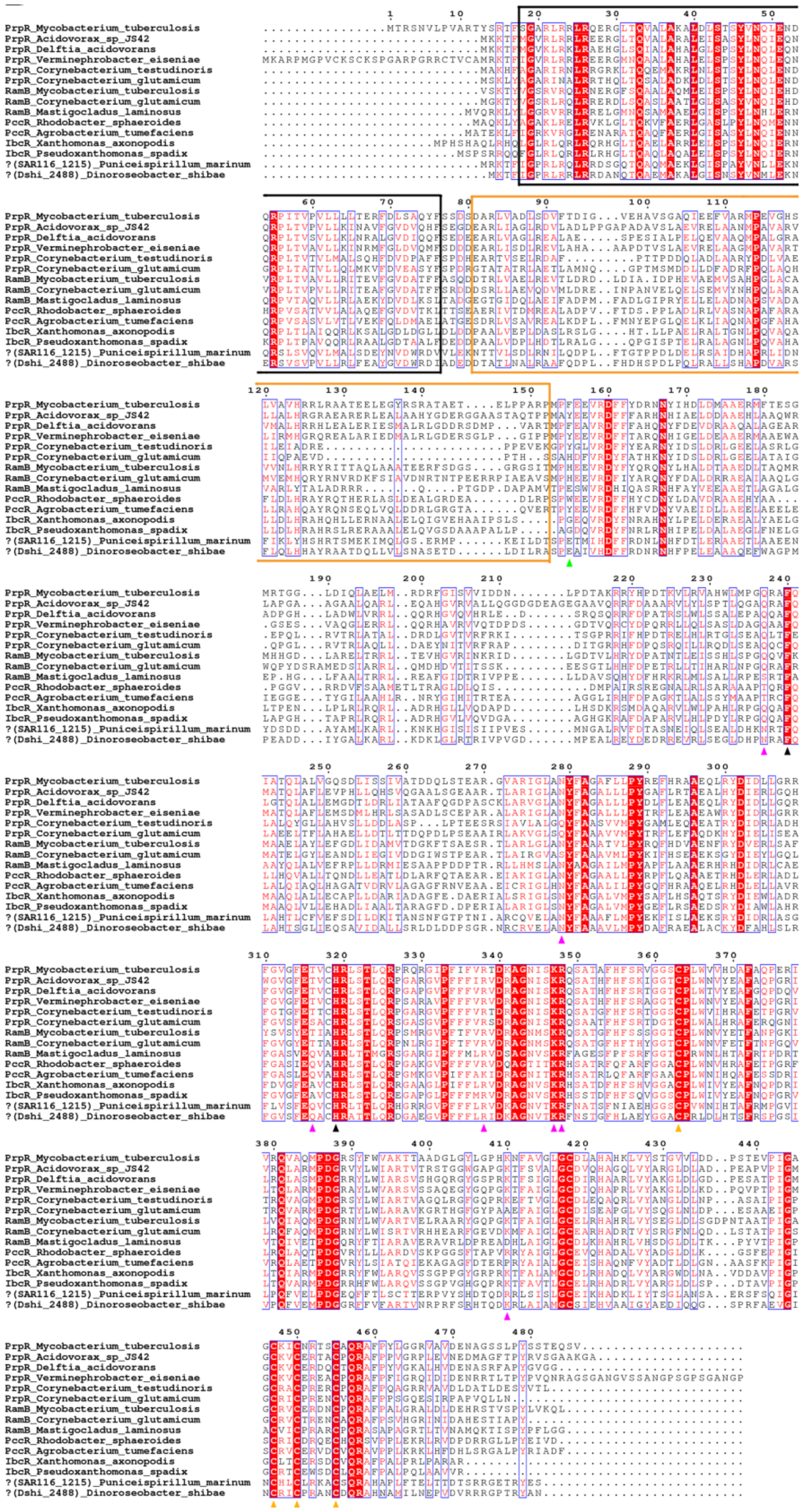

Supplementary Figure S5

Sequence alignment of the protein homologs of MtPrpR. The annotations are based on the crystal structure of MtPrpR: black box, the HTH DNA-binding domain, which is absent in the $MtPrpR_{81\text{-}486}$ construct for crystallization; orange box, the region that is present in the protein construct but not visible in the X-ray crystal structure; green triangle, the residue contacting the acyl group of the CoA derivatives; black triangles, the residues interacting with the adenine group of CoA; purple triangles, the residues forming hydrogen bonds or electrostatic interactions with the CoA; yellow triangles, the residues ligating the [4Fe4S] cluster.

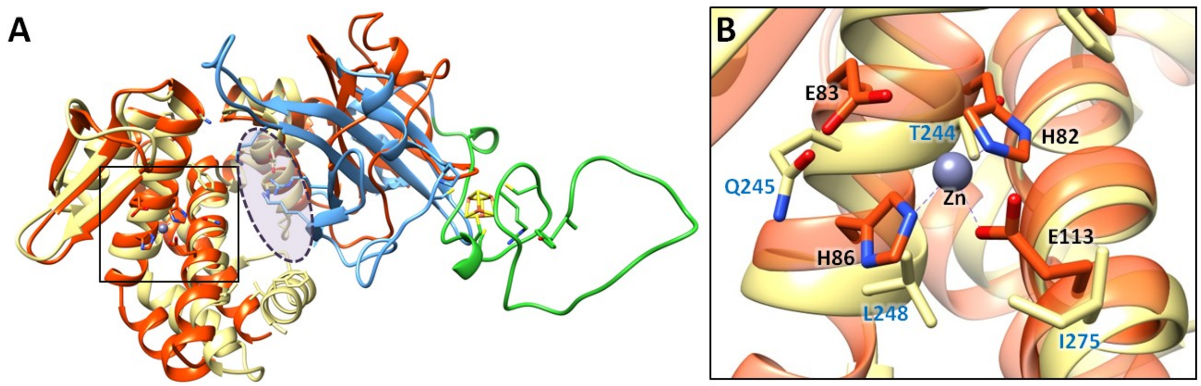

Supplementary Figure S6

(A) Superposition of $MtPrpR_{81\text{-}486}$ (colored by domains as in Figure 1B) and IrrE from *Deinococcus deserti* (orange, PDB accession number: 3DTI). The CoA-binding cavity in $MtPrpR_{81\text{-}486}$ is outlined in dashed ellipse. The zinc-binding site of the putative metaloprotease domain of IrrE is outlined in box. **(B)** Close-up of the zinc-binding site shows that the residues are not conserved in MtPrpR. The IrrE residues are labeled in black; the MtPrpR residues are labeled in blue.

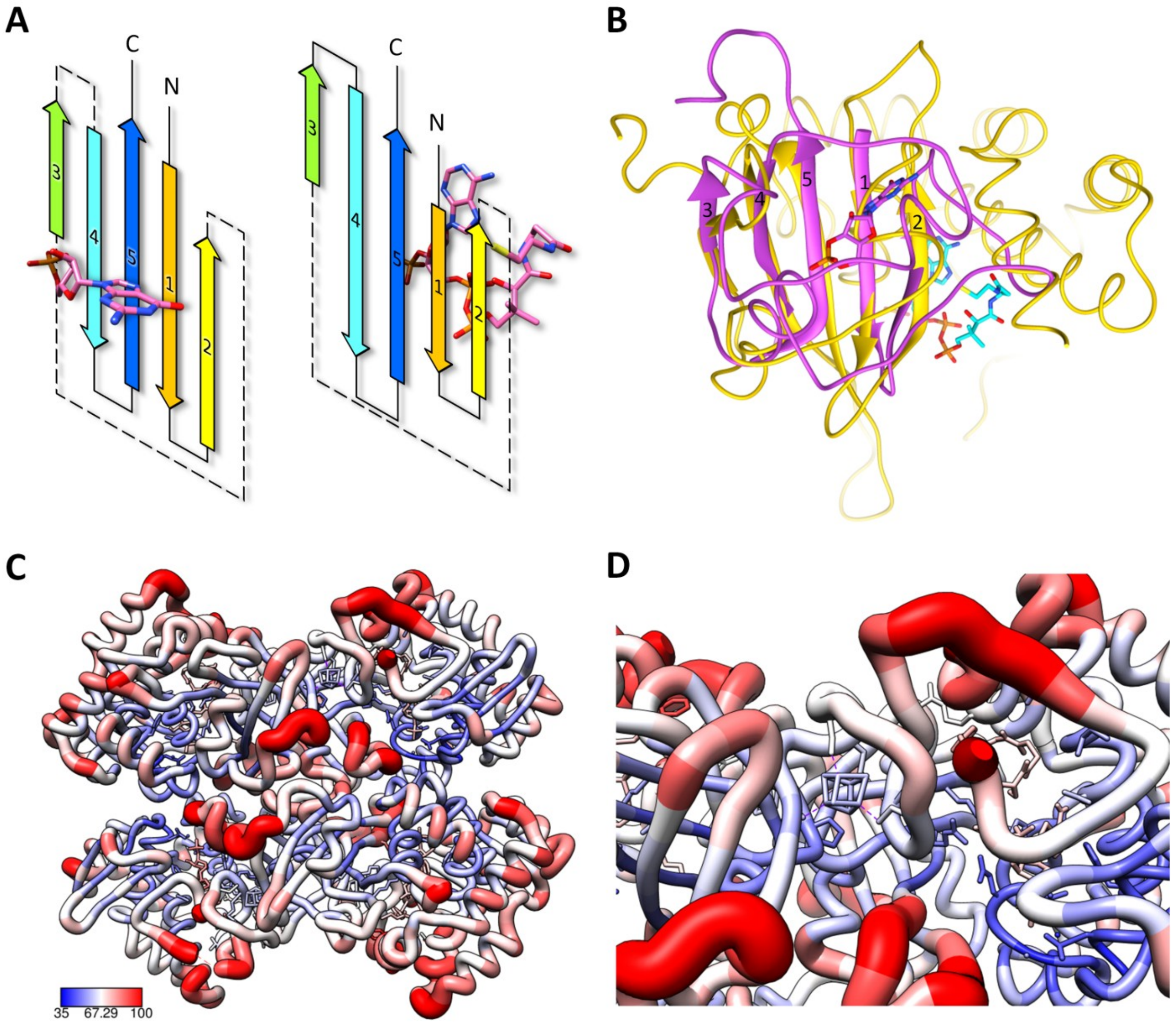

Supplementary Figure S7

(A) Comparison of the ligand binding modes with respect to the central β-sheets between canonical GAF domain (left, cGMP) and the GAF-like domain in MtPrpR (right, CoA). **(B)** Structure comparison between the canonical GAF domain (represented by 1MC0, cGMP bound, both protein and ligand colored in magenta) and GAF-like domain in MtPrpR (protein and ligand colored in yellow and cyan, respectively). **(C)** MtPrpR$_{81\text{-}486}$ backbone colored by B-factors (unit Å^2). **(D)** Close-up of the loop region around the [4Fe4S] cluster in **C.**

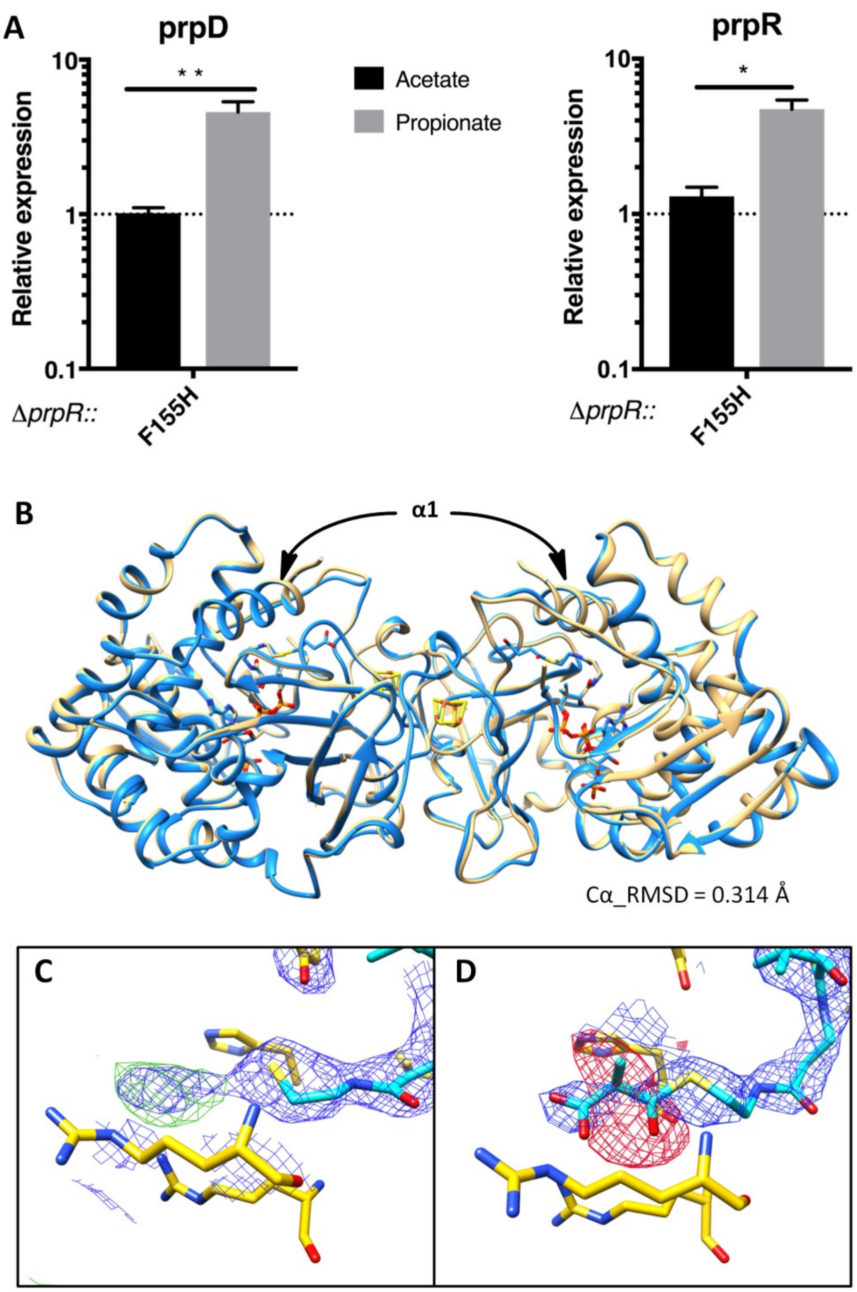

Supplementary Figure S8

(A) Second experiment run of the transcription levels of *prpD* and *prpR* in F155H mutant strain under acetate or propionate carbon sources (experiments identical to the F155H group in Figure 5B). Data presented mean ± SD. *N*=3. Data were analyzed with two-tailed paired sample t-test. *p < 0.0332, **p < 0.0021. **(B)** Structure comparison between $MtPrpR_{81\text{-}486}$_WT and the F155H variant. The first visible helix α1, where residue 155 is located, is indicated (gold, WT; blue, F155H variant). **(C)** 2mFo-DFc map (blue, contoured at 1.0 σ) and positive mFo-DFc map (green, contoured at 3.0 σ) of MtPrpR_F155H with CoA built into the electron density. **(D)** 2mFo-DFc map (blue, contoured at 1.0 σ) and negative mFo-DFc map (red, contoured at 3.0 σ) of MtPrpR_F155H with (S)-methylmalonyl-CoA built into the electron density.

Supplementary Table S1. Primers used in this study

**Cloning**	
Primers	Sequences
PrpR_M1_Fw	5'-GGTCTAGAGGGATCCATGACGCGGAGTAATGTCTTA-3'
PrpR_V9_Fw	5'-CGCGGATCCGTGGCTAGGACGTATTCGAG-3'
PrpR_Rv	5'-GGGGTACCCTCGAGTCAAACCGATTGCTCGGTCGA-3'
PrpR_D81_Fw	5'-TACTTCCAATCCAATGCCGACGCACGGTTGGTGG-3'
PrpR_V486_Rv	5'-TTATCCACTTCCAATGTTAAACCGATTGCTCGGT-3'
PrpR_F155_Fw	5'-TACTTCCAATCCAATGCCTTCGAGGAGGTCCGCGAC-3'
PrpR_E440_Rv	5'-TTATCCACTTCCAATGTTACTCCGTGCTCGGGTCGTC-3'
PrpR_F240A_Fw	5'-CCCGGACAGCGCGCCGCCCAGATCGCCACCCAA-3'
PrpR_F240A_Rv	5'-TTGGGTGGCGATCTGGGCGGCGCGCTGTCCGGG-3'
PrpR_H319A_Fw	5'-TTCGAAACCGTCTGCGCCCGGCTCTCCACACTG-3'
PrpR_H319A_Rv	5'-CAGTGTGGAGAGCCGGGCGCAGACGGTTTCGAA-3'
PrpR_F155H_Fw	5'- GCGCGGCCGATGCCGCACGAGGAGGTCCGCGAC-3'
PrpR_F155H_Rv	5'-GTCGCGGACCTCCTCGTGCGGCATCGGCCGCGC-3'
PrpR_F155A_Fw	5'-GCGCGGCCGATGCCGGCCGAGGAGGTCCGCGAC-3'
PrpR_F155A_Rv	5'-GTCGCGGACCTCCTCGGCCGGCATCGGCCGCGC-3'
PrpR_F155W_Fw	5'-GCGCGGCCGATGCCGTGGGAGGAGGTCCGCGAC-3'
PrpR_F155W_Rv	5'-GTCGCGGACCTCCTCCCACGGCATCGGCCGCGC-3'
PrpR_F155Y_Fw	5'-GCGCGGCCGATGCCGTATGAGGAGGTCCGCGAC-3'
PrpR_F155Y_Rv	5'-GTCGCGGACCTCCTCATACGGCATCGGCCGCGC-3'
PrpR_C363A_Fw	5'-CGGGTCGGCGGCAGCGCCCCGCTGTGGGTGGTC-3'
PrpR_C363A_Rv	5'-GACCACCCACAGCGGGGCGCTGCCGCCGACCCG-3'
PrpR_C450A_Fw	5'-GCGGGCTGCAAGATCGCCAACCGAACGTCGTGC-3'
PrpR_C450A_Rv	5'-GCACGACGTTCGGTTGGCGATCTTGCAGCCCGC-3'
**RT-qPCR for *M. tuberculosis***	
Primers	Sequences
prpR_Fw	5'-ATGTCAACCAGTTGGAGAATGA-3'
prpR_Rv	5'-CGGAATCCGAGGAGAAATACTG-3'
prpD_Fw	5'-TGACTTTCACGACACGTTTCTGGC-3'
prpD_Rv	5'-TGTGGATCTCATAGGCGGTTACCA-3'
icl1_Fw	5'-CAGCACATCCGCACTTTGAC-3'
icl1_Rv	5'-ATCACCACCGTGGGAACATC-3'
ramB_Fw	5'-CCTGCCCGCTGTGGAA-3'
ramB_Rv	5'-GGCGATTTGCACCAAGATCT-3'
sigA_Fw	5'-CAAGTTCTCCACCTACGCTAC-3'
sigA_Rv	5'-GTTGATCACCTCGACCATGT-3'

Supplementary Table S2. Crystallographic data collection and refinement statistics

	SeMet $PrpR_{81-486}$	Native $PrpR_{81-486}$	$PrpR_{81-486}$_F155H	$PrpR_{155-440}$
**PDB code**	6CZ6	6CYY	6CYJ	6D2S
**Data Collection**				
Space Group	$P2_12_12_1$	$P4_12_12$	$P4_12_12$	I222
Unit cell dimensions				
a, b, c (Å)	96.19, 142.37, 145.96	144.87, 144.87, 97.27	144.49, 144.49, 96.19	75.00, 81.72, 95.36
α, β, γ (°)	90.00, 90.00, 90.00	90.00, 90.00, 90.00	90.00, 90.00, 90.00	90.00, 90.00, 90.00
Wavelength (Å)	0.97949	1.07507	1.03328	0.97935
Resolution range (Å)	50.00-2.70 (2.75-2.70)	50.00-2.50 (2.54-2.50)	50.00-2.70 (2.75-2.70)	50.00-1.82 (1.85-1.82)
Unique reflections *	55,505 (2,747)	35,534 (1,773)	27,986 (1,414)	26,651 (1,274)
Multiplicity *	7.4 (7.4)	21.2 (22.6)	17.0 (17.1)	9.5 (7.3)
Completeness (%) *	100.0 (100.0)	98.4 (100.0)	97.8 (100.0)	99.8 (100)
R_{merge} *	0.108 (1.132)	0.059 (0.733)	0.078 (1.052)	0.043 (0.241)
R_{pim} *	0.043 (0.446)	0.013 (0.157)	0.019 (0.258)	0.015 (0.095)
I/σ (I) *	21.80 (1.38)	66.7 (3.77)	45.60 (2.44)	61.53 (10.11)
Wilson B value ($Å^2$)	65.88	70.26	78.74	20.30
**Refinement**				
Resolution (Å)	45.56-2.70 (2.80-2.70)	46.10-2.51 (2.60-2.51)	45.63-2.70 (2.80-2.70)	32.09-1.82 (1.88-1.82)
R_{work}/R_{free} *	0.182/0.212 (0.302/0.345)	0.183/0.214 (0.259/0.307)	0.185/0.218 (0.281/0.364)	0.179/0.214 (0.205/0.269)
No. of atoms	10,515	5,288	5,286	2,484
Protein	10,267	5,172	5,158	2,287
Ligand	224	112	126	5
Solvent	24	4	2	192
Mean B factors ($Å^2$)	67.29	77.75	85.96	27.72
Protein	67.28	77.85	86.05	26.87
Ligand	69.43	73.98	82.36	24.43
Solvent	52.00	62.62	69.59	37.83
Rmsd bond length (Å)	0.005	0.004	0.003	0.007
Rmsd bond angle (°)	0.763	0.823	0.721	0.834
Clashscore	5.90	3.55	6.15	5.67
Number of TLS groups	28	15	15	2
Ramachandran plot (%) (favored/allowed/outliers)	97.69/2.31/0.00	97.87/2.13/0.00	98.47/1.53/0.00	97.79/2.21/0.00
Rotamer outliers (%)	0.94	0.00	0.56	0.00

* Values in parenthesis indicate highest-resolution bin.
